# Insights into the electric double-layer capacitance of two-dimensional electrically conductive metal–organic frameworks†

**DOI:** 10.1039/d1ta04026j

**Published:** 2021-06-25

**Authors:** Jamie W. Gittins, Chloe J. Balhatchet, Yuan Chen, Cheng Liu, David G. Madden, Sylvia Britto, Matthias J. Golomb, Aron Walsh, David Fairen-Jimenez, Siân E. Dutton, Alexander C. Forse

**Affiliations:** Yusuf Hamied Department of Chemistry, University of Cambridge Lensfield Road Cambridge CB2 1EW UK acf50@cam.ac.uk; Department of Chemistry, Imperial College London Exhibition Road London SW7 2AZ UK; The Faraday Institution Quad One, Harwell Science and Innovation Campus Didcot OX11 0RA UK; Cavendish Laboratory, University of Cambridge JJ Thomson Avenue Cambridge CB3 0HE UK; Adsorption & Advanced Materials Laboratory (A^2^ML), Department of Chemical Engineering & Biotechnology, University of Cambridge Philippa Fawcett Drive Cambridge CB3 0AS UK; Diamond Light Source, Harwell Science and Innovation Campus Didcot OX11 0DE UK; Department of Materials, Imperial College London Exhibition Road London SW7 2AZ UK

## Abstract

Two-dimensional electrically conductive metal–organic frameworks (MOFs) have emerged as promising model electrodes for use in electric double-layer capacitors (EDLCs). However, a number of fundamental questions about the behaviour of this class of materials in EDLCs remain unanswered, including the effect of the identity of the metal node and organic linker molecule on capacitive performance, and the limitations of current conductive MOFs in these devices relative to traditional activated carbon electrode materials. Herein, we address both these questions *via* a detailed study of the capacitive performance of the framework Cu_3_(HHTP)_2_ (HHTP = 2,3,6,7,10,11-hexahydroxytriphenylene) with an acetonitrile-based electrolyte, finding a specific capacitance of 110–114 F g^−1^ at current densities of 0.04–0.05 A g^−1^ and a modest rate capability. By directly comparing its performance with the previously reported analogue, Ni_3_(HITP)_2_ (HITP = 2,3,6,7,10,11-hexaiminotriphenylene), we illustrate that capacitive performance is largely independent of the identity of the metal node and organic linker molecule in these nearly isostructural MOFs. Importantly, this result suggests that EDLC performance in general is uniquely defined by the 3D structure of the electrodes and the electrolyte, a significant finding not demonstrated using traditional electrode materials. Finally, we probe the limitations of Cu_3_(HHTP)_2_ in EDLCs, finding a limited stable double-layer voltage window of 1 V and only a modest capacitance retention of 81% over 30 000 cycles, both significantly lower than state-of-the-art porous carbons. These important insights will aid the design of future conductive MOFs with greater EDLC performances.

## Abstract 2

A detailed study into the electric double-layer capacitance of the conductive metal–organic framework Cu_3_(HHTP)_2_ reveals new insights into the capacitive behaviour of this family of materials.

## Graphical abstract

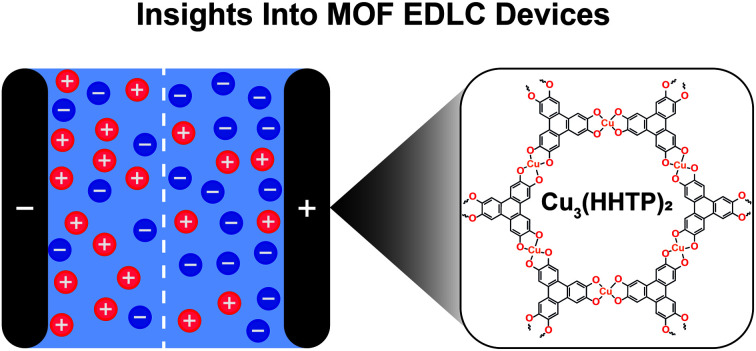

## Introduction

The improvement of energy storage devices is critical for society to meet increasing energy demands and allow for the integration of renewable energy sources into energy grids.^1–3^ Electric double-layer capacitors (EDLCs), a sub-set of supercapacitors, are among the most promising energy storage devices due to their high power densities, which result in rapid charging/discharging times, and excellent cyclabilities. As a result, EDLCs have potential uses in applications where other energy storage devices are not suitable *e.g.*, in heavy electrical vehicles, storing energy rapidly from intermittent renewable energy sources.^3–6^ However, state-of-the-art industrial EDLCs have low energy densities, which impedes their widespread use. Potential performance gains could be achieved by optimising the structure of the electrodes and this may facilitate the use of EDLCs more widely. Structure–property investigations to determine how performance varies with electrode structure are challenging with traditional EDLCs as many use porous carbons as the electrode material.^7,8^ These tend to have poorly defined structures that are difficult to characterise, leading to structure–property investigations with conflicting results.^9–14^

**Fig. 1:**
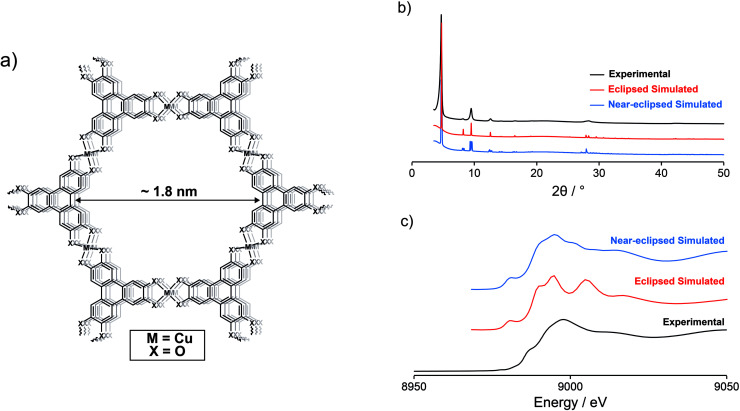
(a) Schematic demonstrating the general structure of hexasubstituted triphenylene-based conductive MOFs. The π–d conjugated 2D sheets stack to form an extended 3D honeycomb structure. This creates pores/channels that run through the material, with a pore size of 1.8 nm as calculated from the simulated structure of Cu_3_(HHTP)_2_. (b) The experimental PXRD pattern of Cu_3_(HHTP)_2_ compares well to simulated PXRD patterns of Cu_3_(HHTP)_2_ with both eclipsed and near-eclipsed crystal structures. (c) Experimentally obtained Cu K-edge XANES of Cu_3_(HHTP)_2_ shows better agreement with the simulated XANES of Cu_3_(HHTP)_2_ with a near-eclipsed crystal structure.

Recently, significant work has been done to develop new electrode materials for EDLCs with well-defined structures. One such class of materials is two-dimensional electrically conductive metal–organic frameworks (MOFs).^15^ These materials are generally formed from the square planar coordination of late transition metal M^2+^ nodes by planar conjugated organic linker molecules to form π–d conjugated 2D sheets. These sheets then stack, normally in an eclipsed or near-eclipsed fashion, to form an extended 3D honeycomb structure, creating pores that run through the material (Fig. 1a).^16,17^ Conductive MOFs are promising for use as EDLC electrodes as they have high intrinsic electrical conductivities (up to 2500 S cm^−1^) and porosities (surface areas of 500 to *ca.* 1400 m^2^ g^−1^), both essential for this function.^18–20^ Furthermore, the tuneable crystalline structures of conductive MOFs make them interesting materials for use as model electrodes in structure–property investigations. Despite this promise and much exploration as electrode materials in other energy storage devices, including batteries, few conductive MOFs have been explored in EDLCs, particularly with more commercially relevant organic electrolytes.^21–26^ However, a key example is Ni_3_(HITP)_2_ (HITP = 2,3,6,7,10,11-hexaiminotriphenylene), which demonstrated high capacitive behaviour (specific capacitance of 111–116 F g^−1^ at 0.05 A g^−1^) as the sole electrode material in a symmetric EDLC with 1 M NEt_4_BF_4_ in acetonitrile electrolyte.^27^ The closely related framework Cu_3_(HHTP)_2_ (HHTP = 2,3,6,7,10,11-hexahydroxytriphenylene) was also explored in supercapacitors with aqueous and solid-state gel electrolytes and, while nanowire arrays (NWAs) of this MOF exhibited good capacitive performance, electrodes made using Cu_3_(HHTP)_2_ powder exhibited relatively poor capacitive behaviour.^28,29^ Here, we build on these studies and present a detailed analysis of the electric double-layer capacitance of Cu_3_(HHTP)_2_ in EDLCs with an organic electrolyte. Using a recently published synthesis, as well as traditional electrode film processing methods, we find that Cu_3_(HHTP)_2_ exhibits very similar performance to Ni_3_(HITP)_2_ in terms of capacitance, rate capability, and cycling stability, suggesting that EDLC performance is independent of the identity of the metal node and organic linker in these almost isostructural frameworks.^30^

## Results and discussion

Cu_3_(HHTP)_2_ was synthesised by modifying a recently published procedure.^30^ The identity and structure of the MOF were confirmed *via* powder X-ray diffraction (PXRD), with the experimentally obtained PXRD pattern comparing well to those simulated using hexagonal eclipsed and monoclinic near-eclipsed crystal structures of Cu_3_(HHTP)_2_ (Fig. 1a, b; ESI Fig. S1, S2 and Table S1†). Both structures are polytypes of the C-centred monoclinic structure due to the sub-supergroup relation but with variations in the stacking of the 2D layers. However, the quality of the PXRD data is insufficient for Rietveld refinement and therefore insufficient to distinguish between the models with any degree of certainty. To gain further information on the structure of the synthesised Cu_3_(HHTP)_2_, Cu K-edge X-ray absorption near edge structure (XANES) was performed on a powdered sample, and the obtained spectrum compared to those simulated using the two crystal structures described above (Fig. 1c). The results are supportive of previous work indicating that Cu_3_(HHTP)_2_ may have a near-eclipsed crystal structure, with a constant stacking shift of the 2D layers, as opposed to the closely related eclipsed structure exhibited by Ni_3_(HITP)_2_.^31^ Cu K-edge XANES was also used to probe the Cu oxidation states present in the MOF. This confirmed that Cu(ii) is the dominant Cu oxidation state in the as-synthesised MOF with no clear evidence for the presence of Cu(i) (ESI Fig. S3†). This result helps to clarify debate in the literature on the Cu oxidation states in the framework, with some previous XANES and X-ray photoelectron spectroscopy (XPS) investigations indicating the presence of Cu(i) in the MOF synthesised using different methods.^32,33^

We subsequently evaluated the porosity and Brunauer-Emmett-Teller (BET) area using 77 K N_2_ adsorption isotherms. A maximum BET area of 794 m^2^ g^−1^ was calculated using Rouquerol's updated criteria implemented in BETSI (ESI Fig. S4†).^34^ This is the highest reported BET area for this material, comparable to that of Ni_3_(HITP)_2_, and confirms permanent porosity, a key requirement for double-layer capacitance.^27,35^ Elemental analysis confirmed that as-synthesised Cu_3_(HHTP)_2_ has approximately the correct stoichiometric ratio of Cu and HHTP. A small amount of a N-containing impurity was also present, most likely due to the use of ammonia as a modulator in the synthesis.

Having characterised the crystalline structure and porosity of Cu_3_(HHTP)_2_, we next examined its electrical conductivity as this is a further key requirement for EDLC electrodes. The electrical conductivity of a pressed pellet of Cu_3_(HHTP)_2_ (two-point probe) was measured as 0.007 S cm^−1^. This is comparable to previously reported values for this MOF (0.0001–0.3 S cm^−1^ for polycrystalline samples).^21,28,31,36^ Composite films of Cu_3_(HHTP)_2_ (85 wt% Cu_3_(HHTP)_2_, 10 wt% carbon black, and 5 wt% PTFE) of *ca.* 250 μm thickness were then prepared by adapting the traditional literature method for the preparation of activated carbon films.^37^ Carbon black was used as a conductive additive to increase the electrical conductivity of the films for use in EDLCs and has negligible contribution to the total capacitance of the cell (ESI Fig. S5†). Films made without the conductive additive (95 wt% Cu_3_(HHTP)_2_ and 5 wt% PTFE) displayed highly resistive behaviour in EDLCs and required very low current densities for analysis, showing the necessity of the conductive additive to achieve good capacitive performance (ESI Fig. S6 and S7†). This indicates a limitation of using this MOF in commercial and model EDLCs. Optimisation of the conductive additive was not performed and may yield further increases in capacitive performance. Interestingly, Cu K-edge XANES on pristine film samples revealed evidence for the presence of Cu(i), with the amount of Cu(i) observed varying between samples (ESI Fig. S8†). Linear combination fitting of this XANES data with standard compounds indicated a maximal Cu(i) content of approximately 20% (ESI Fig. S9 and Table S2†). This underscores the air sensitivity of Cu_3_(HHTP)_2_ and modification of the film-making procedure could be considered in future work if Cu(i) content proves to be problematic.

**Fig. 2:**
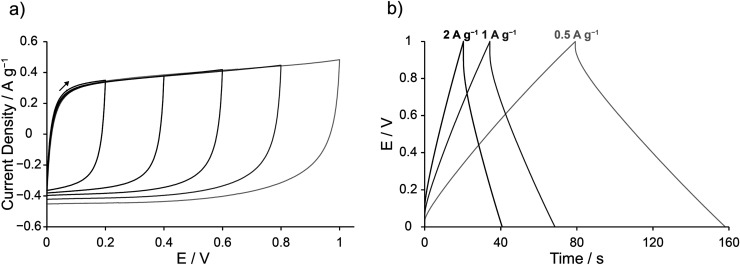
(a) Cyclic voltammograms (CVs) obtained at a scan rate of 10 mV s^−1^ up to 1 V for symmetric EDLCs assembled with Cu_3_(HHTP)_2_ composite film electrodes and 1 M NEt_4_BF_4_ in acetonitrile electrolyte. The black arrow shows the direction of scanning from the start of the scan. (b) Galvanostatic charge–discharge (GCD) profiles at a variety of current densities confirm this behaviour (see labels for current densities).

To investigate the electric double-layer capacitance of Cu_3_(HHTP)_2_, symmetrical EDLCs were assembled using composite Cu_3_(HHTP)_2_ film electrodes and 1 M NEt_4_BF_4_ in acetonitrile electrolyte. Cyclic voltammograms (CVs) and galvanostatic charge–discharge (GCD) experiments on these cells showed nearly rectangular and triangular traces, respectively (Fig. 2), indicative of electric double-layer capacitance. An initial cell voltage window of approximately 1 V, where primarily electric double-layer behaviour was observed, was established for Cu_3_(HHTP)_2_ by running CVs up to progressively higher final voltages. Beyond 1 V, faradaic processes centred at *ca.* 1.1 V were observed (ESI Fig. S10†). This stable voltage window was confirmed by running CVs of Cu_3_(HHTP)_2_ composite electrodes in a three-electrode arrangement with 1 M NEt_4_BF_4_ in acetonitrile. Electric double-layer capacitive behaviour and no faradaic activity were observed for Cu_3_(HHTP)_2_ between the open circuit potential of +0.33 V and −0.27 V *vs.* Ag in the anodic direction, and between the open circuit potential of +0.19 V and +0.79 V *vs.* Ag in the cathodic direction (ESI Fig. S11 and S12†). This is consistent with a working voltage window for Cu_3_(HHTP)_2_ EDLCs of *ca.* 1.0–1.2 V. This sharply contrasts with traditional activated carbons, which have a larger typical working voltage window of *ca.* 2.5 V with this electrolyte, a limitation which is further discussed below.^38^

**Fig. 3:**
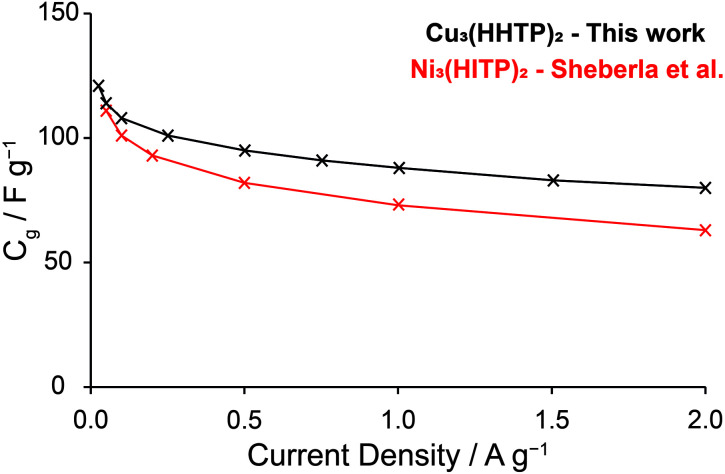
Comparison of specific capacitance *versus* current density graphs for Cu_3_(HHTP)_2_ and Ni_3_(HITP)_2_ (literature).^27^ This demonstrates the similarity in the capacitances of these MOFs in similar symmetric EDLCs. All reported *C*_g_ values are single electrode capacitances calculated from 2-electrode EDLC devices.

To evaluate and compare the capacitive performance of Cu_3_(HHTP)_2_ with other electrode materials, specific capacitance (*C*_g_) was calculated at a variety of current densities from GCD profiles using the Supycap Python code. All reported *C*_g_ values are single electrode values determined from EDLCs by considering the mass of the electroactive electrode material only. At a low current density of 0.04–0.05 A g^−1^, the specific capacitance of Cu_3_(HHTP)_2_ in EDLCs as assembled above was recorded as 110–114 F g^−1^ when charged between 0–1 V (ESI Fig. S13 and Table S3†). This value is very similar to that recorded previously for the almost isostructural framework Ni_3_(HITP)_2_ at a similar current density (111–116 F g^−1^) in EDLCs with 1 M NEt_4_BF_4_ in acetonitrile.^27^ Increasing the current density leads to a decrease in the specific capacitance (Fig. 3), again with very similar results to those reported for Ni_3_(HITP)_2_. Interestingly, these results suggest that the identity of the metal node (Cu or Ni) and ligating heteroatom (O or N) have little/no impact on the double-layer capacitance of these two frameworks. Indeed, Ni_3_(HITP)_2_ and Cu_3_(HHTP)_2_ have very similar 3D structures, with both formed from the eclipsed or near-eclipsed stacking of 2D π–d conjugated layers.^18,31,39^ Therefore, our results suggest high capacitive performance arises from the three-dimensional structures of these MOFs. These results further suggest that the capacitance of an EDLC is uniquely defined by the 3D structure of the electrode and the electrolyte used, although significant further work is needed to confirm this hypothesis by measuring the capacitive performance of a wider range of conducting MOFs with a variety of electrolytes. The equivalent series resistances (*ESR*s) of the EDLCs were measured using both electrochemical impedance spectroscopy (EIS) and GCD profiles, with *ESR*s of between 7–18 Ω obtained for a range of cells (ESI Fig. S14†).

Furthermore, we note higher capacitance retention (79% between 0.25–2 A g^−1^; 72% between 0.25–2.5 A g^−1^) than obtained in previous studies using Cu_3_(HHTP)_2_ powder electrodes in symmetric solid-state EDLCs (30% up to 2 A g^−1^), and capacitance retention on par with that obtained with Cu_3_(HHTP)_2_ NWA electrodes in aqueous (58% up to 2.5 A g^−1^) and solid-state (60% up to 2 A g^−1^) EDLCs.^28,29^ Although a direct comparison with solid-state cells is difficult due to the different phases of the electrolytes, these results illustrate that high capacitive behaviour can be achieved using Cu_3_(HHTP)_2_ powder, which has a simpler synthesis than NWAs, following the addition of a conductive additive (ESI Fig. S15†). However, it must be noted that higher specific capacitances were observed for devices constructed with NWA electrodes (120 F g^−1^ at 0.5 A g^−1^ with a solid-state electrolyte; 195 F g^−1^ at 0.5 A g^−1^ with aqueous electrolyte) than observed in this work.

Another common metric used to compare the EDLC performance of electrode materials is the areal (surface area-normalised) capacitance. In this work, the areal capacitance of Cu_3_(HHTP)_2_ was calculated as approximately 14 μF cm^−2^ at 0.05 A g^−1^. Although this is lower than that reported for Ni_3_(HITP)_2_ (18 μF cm^−2^), significant variation in our values between 14–23 μF cm^−2^ was observed for EDLCs prepared using independent samples of Cu_3_(HHTP)_2_ with different BET surface areas (ESI Table S4†), indicating a potential issue with the reporting of areal capacitances. We also observed variations in the performances of assembled EDLCs as a function of the areal mass loading of the electrodes. In general, EDLCs with higher areal mass loadings exhibited a more rapid decrease in capacitance as a function of current density and a higher resistance than those with lower areal mass loadings (ESI Fig. S13 and Table S3†). This is consistent with previous observations but highlights the need for clear communication on mass loadings when comparing electrode performances.^40^

**Fig. 4:**
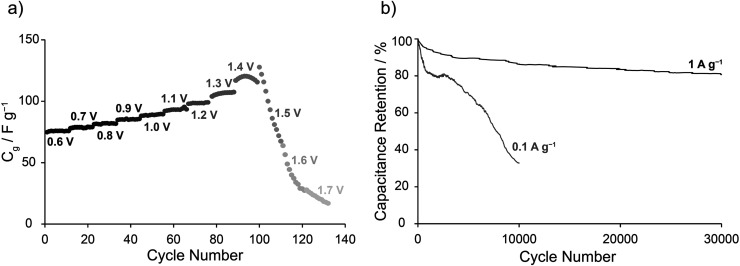
(a) Specific capacitance, calculated from GCD profiles, against cycle number for increasing final cell voltages (see labels). This illustrates the voltage limit of the symmetric Cu_3_(HHTP)_2_ EDLC. All reported *C*_g_ values are single electrode capacitances calculated from 2-electrode EDLC devices. (b) Capacitance retention as a function of cycle number when cycling at 1 A g^−1^ and 0.1 A g^−1^ up to 1 V.

To investigate the suitability of Cu_3_(HHTP)_2_ for both practical supercapacitor applications and structure-property investigations, the voltage limits and cycling stability were studied in more detail. To probe the voltage limits of the cell, GCD experiments at a current density of 0.1 A g^−1^ were run with increasing final cell voltages from 0.6 V until the failure of the cell was observed. This showed an initial consistent increase in the specific capacitance with increasing final voltage followed by a rapid decrease upon cycling beyond 1.3 V (Fig. 4a). This demonstrates that the voltage limit of Cu_3_(HHTP)_2_ in a symmetric EDLC is approximately 1.3 V under these charging/discharging conditions, beyond which rapid degradation of the Cu_3_(HHTP)_2_ electrodes occurs, causing irreversible loss in capacitance. Rapid capacitance loss when cycling above this cell voltage was confirmed *via* CV experiments cycling up to a cell voltage of 1.6 V (ESI Fig. S16†). Degradation was confirmed by examining the Cu K-edge XANES of Cu_3_(HHTP)_2_ composite electrodes from an EDLC held at a cell voltage of 1.5 V for 1 h (ESI Fig. S17†). A shift of the absorption edge to a lower energy, in addition to the appearance of an inflection at *ca.* 8981 eV, indicate formation of Cu(i) in the negative electrode. In the positive electrode, the appearance of the feature at *ca.* 8981 eV indicates a significant change in the coordination environment around Cu to a lower symmetry environment. The shift of the rising edge to higher energies suggests an oxidation process may also occur in the positive electrode. These results indicate fundamental changes to the MOF structure in both electrodes and hint at potential degradation mechanisms, although further work is required to study these processes in more detail.

To further explore the stable working voltage window of Cu_3_(HHTP)_2_ EDLCs, Cu K-edge XANES studies were carried out on electrodes extracted from EDLCs held at different cell voltages for a period of 1 h (ESI Fig. S18†). For a cell voltage of 0.5 V, minimal changes were observed in the XANES spectra. However, for a cell voltage of 0.8 V, the XANES data suggest structural changes to Cu_3_(HHTP)_2_ in the positive electrode. This suggests that kinetically slow faradaic processes may occur at cell voltages below 1.1 V but are missed due to the scan rates used in the above electrochemistry experiments (Fig. 2). This hypothesis was confirmed by obtaining a CV at a scan rate of 0.1 mV s^−1^ up to 1 V, with faradaic activity observed at this slow scan rate upon cycling past 0.8 V (ESI Fig. S19†). This highlights that Cu_3_(HHTP)_2_ may only be kinetically stable up to 1 V, a possible limitation that is explored further below.

Finally, the cycling stability of symmetric Cu_3_(HHTP)_2_ EDLCs was investigated at two different current densities in GCD experiments limited to a maximum cell voltage of 1 V. Reasonable cycling stability was observed when cycled between 0–1 V at 1 A g^−1^, with capacitance retention of 81% over 30 000 cycles (Fig. 4b). The capacitance retentions after 5000 and 10 000 cycles (90% and 86%, respectively) compare well with those of Ni_3_(HITP)_2_, approx. 90% over 10 000 cycles, and Cu_3_(HHTP)_2_ NWA devices with an aqueous electrolyte, 79.9% over 5000 cycles (ESI Fig. S20 and S21†).^27,29^ This further highlights the similarities in capacitive performance between Ni_3_(HITP)_2_ and Cu_3_(HHTP)_2_, and is further evidence that electrodes manufactured from Cu_3_(HHTP)_2_ powder can achieve high EDLC performance on par with those made with Cu_3_(HHTP)_2_ NWAs. Cu K-edge XANES showed minimal changes to the edge position and pre-edge peaks following this cycling, confirming the stability of Cu_3_(HHTP)_2_ upon extensive cycling at this current density (ESI Fig. S22†).

The capacitance retention of Cu_3_(HHTP)_2_ EDLCs in this work, however, was significantly lower when cycled at a lower current density of 0.1 A g^−1^, with only 32% capacitance retention after 10 000 cycles (Fig. 4b). Cu K-edge XANES of the positive electrode following this cycling again provided evidence for a change in the MOF structure, confirming degradation at this current density and further emphasising that Cu_3_(HHTP)_2_ is only kinetically stable when cycled between 0–1 V (ESI Fig. S23†). This is the first work to highlight the difference in capacitance retention at different current densities with this family of conducting frameworks. These findings raise questions about the practical applicability of these frameworks in commercial devices. Future studies to identify the degradation mechanisms in these frameworks may allow for the design of conductive MOFs with wider double-layer stability windows, and thus improved capacitive performances. Varying the metal node or organic linker molecule may be a viable method to increase the double-layer potential window.^41^

Furthermore, the capacitance retention of Cu_3_(HHTP)_2_ is significantly lower than that of YP50F, a commercial microporous activated carbon, when cycled in an EDLC with 1 M NEt_4_BF_4_ in acetonitrile. In our work, YP50F exhibited a capacitance retention of 99% over 10 000 cycles when cycled between 0–2.5 V at 2 A g^−1^ (ESI Fig. S24†). This illustrates that, while this family of MOFs have specific and areal capacitances on par or exceeding current state-of-the-art carbons (YP50F displays a specific capacitance of *ca.* 90–100 F g^−1^ in this system), significant improvement is required to achieve comparable cycling stability. Cu_3_(HHTP)_2_ displayed additional limitations relative to YP50F with the same organic electrolyte. As noted previously, YP50F has a larger working double-layer voltage window compared to Cu_3_(HHTP)_2_ (*ca.* 2.5 V *vs. ca.* 1 V), leading to a higher energy density and greater overall charge storage. In addition, the rate capability of YP50F is significantly higher than that of Cu_3_(HHTP)_2_, with 94% capacitance retention between 0.5–10 A g^−1^. This allows for higher current densities to be used, resulting in faster charging and discharging times (ESI Fig. S25†). This is the first work to call attention to these key differences and illustrates major disadvantages of using this family of conductive MOFs in EDLCs instead of activated carbons, as well as raising questions about its suitability as a model electrode material in structure–property investigations.

## Conclusions

We have demonstrated that the conductive MOF Cu_3_(HHTP)_2_ displays good capacitive behaviour in symmetric EDLCs with 1 M NEt_4_BF_4_ in acetonitrile, with a specific capacitance of 110–114 F g^−1^ at 0.04–0.05 A g^−1^ recorded. Our work shows that the previously observed capacitive behaviour of Ni_3_(HITP)_2_ is not unique amongst layered conducting MOFs and has expanded the family of conductive MOFs which is known to display capacitive performance in EDLCs with organic electrolytes. Notably, Cu_3_(HHTP)_2_ can be synthesised using all commercially available starting materials, and we have demonstrated that standard electrode fabrication techniques using Cu_3_(HHTP)_2_ powder can be employed with this framework to achieve good capacitive performance, making this MOF an accessible model system for further study. The similarity in the specific capacitances of Cu_3_(HHTP)_2_ and Ni_3_(HITP)_2_ at low current densities with the same organic electrolyte indicates that the capacitive performance may be independent of the identity of the metal node and organic linker molecule for these two nearly isostructural frameworks. Importantly, this further suggests that the capacitive performance of an EDLC more generally may be uniquely defined by the 3D structure of the electrodes and the electrolyte. However, significant additional work is needed to confirm these hypotheses. Finally, our work also illustrates several limitations of using current conductive MOFs in EDLCs, notably the significantly lower cycling stability, stable double-layer voltage window, and rate capability relative to state-of-the-art carbon materials. This raises questions about the practical applicability of these frameworks in commercial devices. Ultimately, our work will guide the design of next generation metal–organic frameworks with improved energy storage performance.

## Experimental section

### Materials

Starting materials were purchased from Sigma-Aldrich and used without modification unless stated. Ethanol was purchased from VWR International. Aqueous ammonia (35%) solution and acetone were purchased from Fisher Scientific. YP50F was purchased from Kuraray. Acetylene black carbon (surface area = 75 m^2^ g^−1^) was purchased from Alfa Aesar. 2,3,6,7,10,11-hexahydroxytriphenylene hydrate (H_6_HHTP·*x*H_2_O) was purchased from TCI. Tetraethylammonium tetrafluoroborate (NEt_4_BF_4_) was dried under vacuum at 100 °C for 48 h before transferring to a N_2_-filled glovebox. Anhydrous acetonitrile was purged with N_2_ for 3 h before taking it into a N_2_-filled glovebox, where it was further dried by the addition of activated 3 Å molecular sieves. Sieves were activated at 250 °C in a vacuum oven for 12 h prior to transferring into a N_2_-filled glovebox.

### Synthesis of Cu_3_(HHTP)_2_

Cu_3_(HHTP)_2_ was synthesised by modifying a recently published literature procedure.^30^ A solution of Cu(NO_3_)_2_·3H_2_O (0.127 g, 0.526 mmol, 1.65 eq.) and aqueous ammonia (35%) solution (0.829 mL, 15.0 mmol, 47 eq.) in distilled water (2 mL) was prepared. The resulting royal blue solution was added dropwise to a dispersion of 2,3,6,7,10,11-hexahydroxytriphenylene hydrate, H_6_HHTP·*x*H_2_O, (0.103 g, 0.318 mmol, 1.00 eq.) in distilled water (8.4 mL). The resulting mixture was heated in a furnace oven at 80 °C for 24 h in a 40 mL screw vial (Thermo Scientific; B7999-6), closed with a screw cap fitted with a septum as a safety precaution in the event of over pressurisation. The dark blue precipitate formed was separated by centrifugation and the supernatant layer was discarded. The dark blue precipitate was then washed successively with water (3 × 30 mL), ethanol (4 × 30 mL), and acetone (4 × 30 mL). Washing was performed by centrifuging the precipitate with the desired washing solvent for 15–30 minutes before removing the supernatant layer and replacing with fresh washing solvent. No soaking of the precipitate was performed. The precipitate was then filtered with vacuum filtration and the resulting dark blue powder was dried at 75 °C under dynamic vacuum for 72 h and then stored in a N_2_-filled glovebox until used.

We found that rapid washing (completed in *ca.* 5 h) and activation of the synthesised Cu_3_(HHTP)_2_ to minimise its exposure to air was required to ensure a high porosity and a wider stable double-layer voltage window.

### Elemental analysis

Laboratory elemental analysis was performed on Cu_3_(HHTP)_2_ as synthesised above by the Microanalysis Facility at the Yusuf Hamied Department of Chemistry, Cambridge.

Cu content was determined *via* inductively coupled plasma optical emission spectroscopy (ICP-OES) using a Thermo Scientific iCAP-7400 ICP spectrometer. 1.3610 mg of Cu_3_(HHTP)_2_ was digested in 5 mL of concentrated HNO_3_ (67–69%, trace metal, Fisher Scientific), and the sample diluted with 5 mL of water. A 0.5 mL aliquot was then diluted to 10 mL with water. Cu concentration of the resulting solution was determined using calibration curves constructed from standard solutions (multi-element standard solution for ICP IV, Fisher Scientific). C, H and N content was determined *via* CHN combustion analysis using an Exeter Analytical CE-440, with combustion at 975 °C.

Calculated for Cu_3_(HHTP)_2_: Cu, 23.1 wt%; C, 52.3 wt%; H, 1.5 wt%.

Experimental results for Cu_3_(HHTP)_2_ synthesised above: Cu, 21.7 wt%; C, 48.9 wt%; H, 2.4 wt%; N, 2.8 wt%.

These results confirm that the as-synthesised Cu_3_(HHTP)_2_ has approximately the correct stoichiometric ratio of Cu and HHTP. It also indicates the presence of a N-containing impurity leftover in the MOF following washing.

### X-ray diffraction

Laboratory powder X-ray diffraction data were collected on a Malvern Panalytical Empyrean instrument, equipped with an X'celerator Scientific detector using non-monochromated Cu K_α_ radiation (*λ* = 1.5418 Å). Borosilicate glass capillary tubes (0.5 mm outside diameter, 0.01 mm wall thickness; Capillary Tube Supplies Ltd.) were loaded with the sample in a N_2_-filled glovebox, with NiCr wire used to aid packing. The capillary was then sealed in the N_2_-filled glovebox using EA 3430 epoxy adhesive (Loctite), which was allowed to cure for 5 h before removing the capillary from the glovebox. The data were collected at room temperature over a 2*θ* range of 3–50°, with an effective step size of 0.017° and a total collection time per scan of 1 h. Multiple scans were chosen to minimise the possibility of saturating the detector as well as to detect any possible changes with time (none were observed). The presented experimental PXRD is a sum average of 15 scans.

Simulated PXRD patterns were produced using GSAS-II Crystallography Data Analysis Software.^42^ Computational structures used to produce the simulated PXRD patterns and XANES are available at: https://doi.org/10.5281/zenodo.4694845

### Gas adsorption measurements

Low pressure N_2_ isotherms (adsorption and desorption) were collected using a Micromeritics 3Flex at 77 K. Prior to analysis, samples were degassed in a Schlenk flask at 80 °C for 24 h. *In situ* degassing (80 °C, 24 h) was further performed on a Micromeritics VacPrep. Material BET areas were calculated from the isotherms using the BET equation and Rouquerol's consistency criteria implemented in BETSI.^32,43^ The micropore volume (*W*_0_) and the total (*V*_tot_) pore volumes were calculated at *P*/*P*_0_ of 0.1 and 0.99, respectively. For Cu_3_(HHTP)_2_, a Type I N_2_ isotherm was observed, with high gas uptake below 0.1 *P*/*P*_0_ indicating extensive microporosity. See the ESI Appendix for full BETSI readouts.†

### Conductivity measurements

The electrical conductivity of Cu_3_(HHTP)_2_ samples was measured *via* a two-point probe method using a homemade set-up. Samples were pressed between two stainless-steel electrodes using a hydraulic press (Specac). Insulating PTFE disks were used to prevent a short circuit through the press. All measurements were conducted with a loading of between 1.50–1.57 ton-force cm^−2^. Resistances were measured using a Keithley 2000 Multimeter.

The conductivity, *σ* (S cm^−1^), of the sample was calculated according to: *σ* = *L*/*RA*, where *L* is the thickness of the sample (cm), *A* is the area of the sample (cm^2^), and *R* is the measured resistance (Ω). All values of *L* and *A* were measured following completion of the measurement, assuming a non-elastic material. Based on multiple measurements of the resistance and the thickness of the sample, the error on the calculated conductivity value is *ca.* ±6.8%.

Pellets composed of Cu_3_(HHTP)_2_ were prepared by loading the material into a 13 mm evacuable pellet die (Specac) and applying a force of 3 ton-force cm^−2^ for 5 min with a hydraulic press (Specac). The areal mass loading of the pellets was approximately 50 mg cm^−2^. The thickness of the pellets was measured using a digital micrometer (Mitutoyo) as approximately 330 μm.

### Electrode film preparation

Freestanding composite MOF films were prepared by adapting the traditional literature method for activated carbons.^11^ Cu_3_(HHTP)_2_ powder and acetylene black were lightly ground together in a vial before ethanol (*ca.* 1.5 mL) was added to produce a loose slurry. This was sonicated for 15 min before being added to PTFE dispersion (60 wt% in water) in a few drops of ethanol in a watch glass. The slurry was stirred by hand in the watch glass for 40 min under ambient conditions. The film was gradually formed upon drying of the slurry before being transferred to a glass surface, where it was kneaded for 20 min to ensure homogenous incorporation of the active materials and PTFE and then rolled into a freestanding film using a homemade aluminium rolling pin. The film was dried *in vacuo* at 75 °C for at least 48 h to remove any remaining ethanol. The masses of components were calculated so that the final film had a composition of 85 wt% Cu_3_(HHTP)_2_, 10 wt% acetylene black, and 5 wt% PTFE.

Freestanding acetylene black, YP50F, and Cu_3_(HHTP)_2_ films were prepared using the same technique. These had a final composition of 95 wt% electroactive material and 5 wt% PTFE.

### EDLC assembly

Symmetric electric double-layer capacitors (EDLCs) with Cu_3_(HHTP)_2_ composite and acetylene black film electrodes were prepared in Swagelok PFA-820-6 union tube fittings with homemade stainless-steel plugs as current collectors. Electrodes were cut from freestanding films in a N_2_-filled glovebox using a ¼’’ stainless-steel manual punching cutter (Hilka Tools), with areal mass loadings ranging between 10–35 mg cm^−2^. An excess of 1 M NEt_4_BF_4_ in anhydrous acetonitrile was used as an electrolyte. This solution was prepared in a N_2_-filled glovebox. Whatman glass microfiber filter (GF/A), cut with a ⅜’’ stainless-steel manual punching cutter, was used as a separator. This was dried *in vacuo* at 100 °C for 24 h prior to use. EDLCs were hand-sealed until air-tight before being removed from the glovebox for electrochemical testing.

Symmetric electric double-layer capacitors (EDLCs) with YP50F film electrodes were prepared as coin cells in CR2032 SS316 coin cell cases (Cambridge Energy Solutions). Electrodes were cut from freestanding YP50F films with areal mass loadings ranging between 10–15 mg cm^−2^. The electrodes were dried *in vacuo* at 100 °C for at least 24 h prior to assembling the cell in a N_2_-filled glovebox. A 1 M solution of NEt_4_BF_4_ in anhydrous acetonitrile was used as an electrolyte. This solution was prepared in a N_2_-filled glovebox. Whatman glass microfiber filter (GF/A) was used as a separator. This was dried *in vacuo* at 100 °C for 24 h prior to use. Each coin cell contained two SS316 separator disks and one SS316 spring to ensure sufficient pressure in the cell. The coin cells were sealed in the glovebox using a Compact Hydraulic Coin Cell Crimper (Cambridge Energy Solutions).

Cu_3_(HHTP)_2_ composite cells were assembled in Swagelok PFA-820-6 union tube fittings as opposed to in CR2032 SS316 coin cell cases (Cambridge Energy Solutions) as the disassembly of the cell, without inadvertently causing cell discharge, was easier with the tube fittings.

### Three-electrode cell assembly

Three-electrode cells were prepared in Swagelok PFA-820-3 union tube fittings with homemade stainless-steel plugs as current collectors. Cu_3_(HHTP)_2_ composite electrodes with areal mass loadings ranging between 12–20 mg cm^−2^ were used as working electrodes. Overcapacitive YP50F activated carbon film electrodes with areal mass loadings of 35–40 mg cm^−2^ were used as counter electrodes. Ag wire was used as a pseudo-reference electrode. A 1 M solution of NEt_4_BF_4_ in anhydrous acetonitrile was used as an electrolyte. Whatman glass microfiber filter (GF/A) was used as a separator. All measurements were performed under dry and oxygen-free conditions in a N_2_-filled glove box. Under these conditions, the ferrocene–ferricenium (Fc/Fc^+^) redox couple was measured at 0.63 ± 0.01 V *versus* Ag. All potentials discussed for the three-electrode cell are referenced to Ag.

### Electrochemical characterisation

All electrochemical measurements were carried out using Biologic SP-150 and VSP-3e potentiostats and a Biologic BCS-800 series ultra-precision battery cycler. Electrochemical impedance spectroscopy (EIS) measurements were performed in the frequency range from 200 kHz to 3–10 mHz using a single-sinusoidal signal with a sinus amplitude of 10 mV. No drift correction was applied. The specific capacitance, *C*_g_ (F g^−1^), was calculated from galvanostatic charge–discharge (GCD) discharge profiles using the Supycap Python code. *C*_g_ values were determined using only the mass of active material (i.e., Cu_3_HHTP_2_) in the EDLCs.

The equivalent series resistance (*ESR*) was calculated from both Nyquist plots (produced from EIS measurements) and from the voltage drop at the beginning of GCD discharge profiles. For the calculation from Nyquist plots, the *ESR* was obtained from extrapolation of the low frequency response onto the real (Re(*Z*)) axis, as is consistent with the literature.^44^ For the calculation from GCD discharge profiles, the Supycap Python code was used.

Current densities were calculated by dividing the current applied during the GCD experiment, *I*, by the average mass of active material per electrode, *m̄*.

For full details of the calculations and methods used in the Supycap Python code, please visit: https://github.com/AdaYuanChen/Supycap

### X-ray absorption spectroscopy

Cu K-edge X-ray absorption near edge structure (XANES) measurements were performed at the B18 beamline at Diamond Light Source. Measurements at the Cu K-edge were recorded in fluorescence yield mode. Energy calibration was done with Cu metal as a reference. XANES data were processed and analysed using the Athena program of the Demeter software package.^45^

XANES electrode samples were prepared from the disassembly of EDLC cells. The cells were disassembled in a N_2_-filled glovebox and the electrodes were isolated and packaged into air-tight foil/poly pouches (Sigma-Aldrich). Cu(i) standard (Cu_2_O, CuOAc) and Cu_3_(HHTP)_2_ powder samples were prepared by grinding a small amount (*ca.* 5 wt%) of the standard with cellulose in a N_2_-filled glovebox before packaging into an air-tight foil/poly pouch. Cu(ii) standard samples (CuO, Cu(OAc)_2_) were prepared by grinding a small amount of the standard (*ca.* 5 wt%) with cellulose in ambient conditions before pressing into a pellet using a hydraulic press (Specac) as described previously.

In this work, the edge is defined as the energy at normalised *xμ*(*E*) = 0.5.

XANES calculations were done using the FEFF 9.0 code.^46,47^ The Full Multiple Scattering (FMS) and Self Consistent Field (SCF) radii were set to 8.0 Å and 7.5 Å respectively and calculations were done using the Hedin–Lundqvist exchange-correlation potential. The exchange potential was offset by 2 eV to account for errors in the calculated Fermi level, and an imaginary energy of 0.5 eV was added to correct for instrumental broadening. All other FEFF parameters were set to the default values. A red shift of the simulated spectra was required to align it with the experimental spectrum.

## Data availability

The PXRD, gas sorption, XANES, elemental analysis, conductivity measurement, and electrochemistry data are available in the Cambridge Research Repository, Apollo, with the identifier DOI: 10.17863/CAM.71950.

## Author contributions

J. W. G. and A. C. F. designed the research. J. W. G and C. J. B. performed the material synthesis and electrode film fabrication. M. J. G. performed the crystal structure modelling. J. W. G., C. J. B. and M. J. G. performed and interpreted the PXRD measurements. D. G. M. performed and interpreted the N_2_ gas adsorption measurements. J. W. G. and C. L. performed and interpreted the conductivity measurements. J. W. G. performed the electrochemical cell assemblies. J. W. G. and Y. C. interpreted the electrochemical cell characterisation measurements. S. B. and J. W. G. interpreted the XANES measurements. S. B. performed and interpreted the XANES modelling. All authors interpreted the results and contributed to the writing of the manuscript.

## Conflicts of interest

There are no conflicts to declare.

## Supplementary Material

TA-009-D1TA04026J-s001
