# Supplementary material for: Insights into the electric double-layer capacitance of two-dimensional electrically conductive metal–organic frameworks
Source: J Mater Chem A Mater. 2021 Jun 25;9(29):16006–15. doi: 10.1039/d1ta04026j (PMC8315177; doi:10.1039/d1ta04026j)
Supplement: TA-009-D1TA04026J-s001 [file TA-009-D1TA04026J-s001.pdf]

Electronic Supplementary Material (ESI) for Journal of Materials Chemistry A.
This journal is © The Royal Society of Chemistry 2021

Supplementary Information for:

Insights into the electric double-layer capacitance of two-dimensional electrically conductive metal-organic frameworks

Jamie W. Gittins[1], Chloe J. Balhatchet[1], Yuan Chen[1, 2, 3], Cheng Liu[4], David G. Madden[5], Sylvia Britto[6], Matthias J. Golomb[7], Aron Walsh[7], David Fairen-Jimenez[5], Siân E. Dutton[4], Alexander C. Forse[1]*

[1] Yusuf Hamied Department of Chemistry, University of Cambridge, Lensfield Road, Cambridge CB2 1EW, U.K.

[2] Department of Chemistry, Imperial College London, Exhibition Road, London SW7 2AZ, U.K.

[3] The Faraday Institution, Quad One, Harwell Science and Innovation Campus, Didcot OX11 0RA, U.K.

[4] Cavendish Laboratory, University of Cambridge, JJ Thomson Avenue, Cambridge CB3 0HE, U.K.

[5] Adsorption & Advanced Materials Laboratory (A^{2}ML), Department of Chemical Engineering & Biotechnology, University of Cambridge, Philippa Fawcett Drive, Cambridge CB3 0AS, U.K.

[6] Diamond Light Source, Harwell Science and Innovation Campus, Didcot OX11 0DE, U.K.

[7] Department of Materials, Imperial College London, Exhibition Road London SW7 2AZ, U.K.

*acf50@cam.ac.uk

Contents

Additional Discussion **3**
Cu K-edge XANES Results 3
Figure S1 .... **4**
Figure S2 .... **4**
Figure S3 .... **5**
Figure S4 .... **6**
Figure S5 .... **7**
Figure S6 .... **8**
Figure S7 .... **8**
Figure S8 .... **9**
Figure S9 .... **10**
Figure S10 .... **10**
Figure S11 .... **11**
Figure S12 .... **12**
Figure S13 .... **13**
Figure S14 .... **13**
Figure S15 .... **14**
Figure S16 .... **14**
Figure S17 .... **15**
Figure S18 .... **16**
Figure S19 .... **17**
Figure S20 .... **18**
Figure S21 .... **18**
Figure S22 .... **20**
Figure S23 .... **19**
Figure S24 .... **20**
Figure S25: **19**
Table S1: Crystallographic Data for Simulated $Cu_3(HHTP)_2$ Structures **21**
Table S2: Cu K-edge XANES Linear Combination Fittings **22**
Table S3: Performance Comparison of Three Independent EDLCs **23**
Table S4: Comparison of Areal Capacitance of EDLCs Made from Different Batches of $Cu_3(HHTP)_2$ **24**
Appendix **25**

Additional Discussion

Cu K-edge XANES Results

The $Cu_3(HHTP)_2$ powder and pristine electrode samples exhibit a peak at ca. 8978 eV due to the 1s → 3d transition of Cu(II).[1, 2] As this is a dipole forbidden transition, the intensity is very low. In Cu(II) compounds the 1s → 4p transition is observed at higher energies (ca. 8986 eV), as is also seen in the powder and pristine electrode samples.[2]

Cu(I) complexes are generally characterized by the appearance of a 1s → 4p transition at ca. 8981 eV.[3, 4] The negative electrode from the EDLC held at a cell voltage of 1.5 V for 1 h exhibits a shift of the absorption edge to lower energies as well as exhibiting an inflection at ca. 8981 eV, indicating the formation of Cu(I) (Fig. S17). This is consistent with the expectation of a reduction process occurring in the negative electrode.

At first glance, the prominent peak at ca. 8982 eV in the positive electrode from the EDLC held at a cell voltage of 1.5 V appears to be due to a 1s → 4p transition of a reduced Cu(I) (Fig. S17). However, the shift of the rising edge to higher energies is suggestive of an oxidation process occurring. It is known that Cu(III) compounds exhibit a pre-edge feature at ca. 8981 eV due to a shift of the 1s → 3d transition to higher energies, so this is a possibility although further calculations are required to confirm this.[5] Irrespective of the oxidation state of Cu, the appearance of the feature at ca. 8981 eV in the positive electrode indicates a significant change in coordination environment around the Cu nodes.[4]

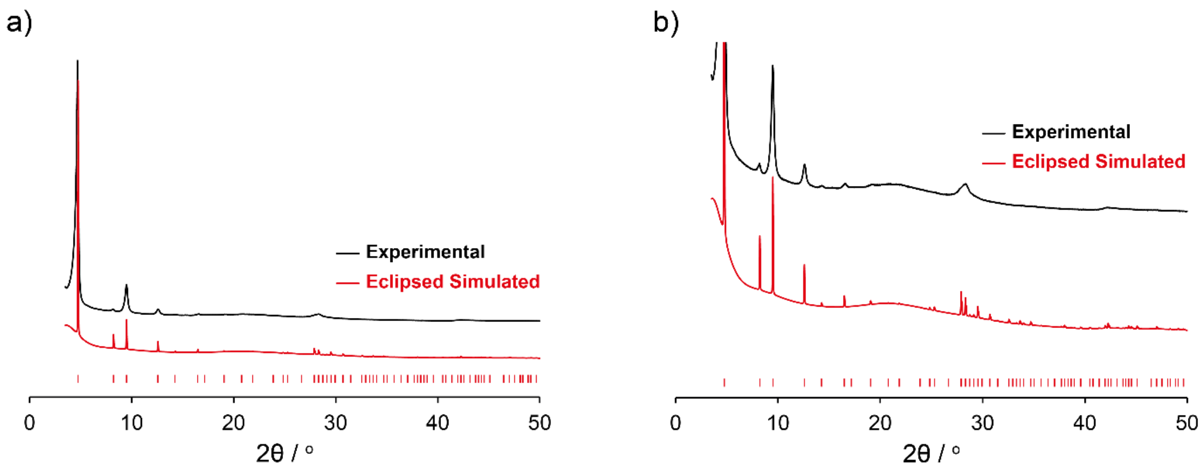

Figure S1: a) Comparison of the experimentally obtained PXRD pattern from $Cu_3(HHTP)_2$ synthesised in this work with the simulated PXRD pattern from $Cu_3(HHTP)_2$ with an eclipsed primitive hexagonal crystal structure (space group = P622), in which the 2D layers of $Cu_3(HHTP)_2$ are eclipsed. Note that the amorphous peak at $2\theta \approx 21$ ° is likely due to the borosilicate glass capillary tube.[6] b) A zoomed in view of the comparison for better visibility. Note that this data was of insufficient quality for a Rietveld refinement.

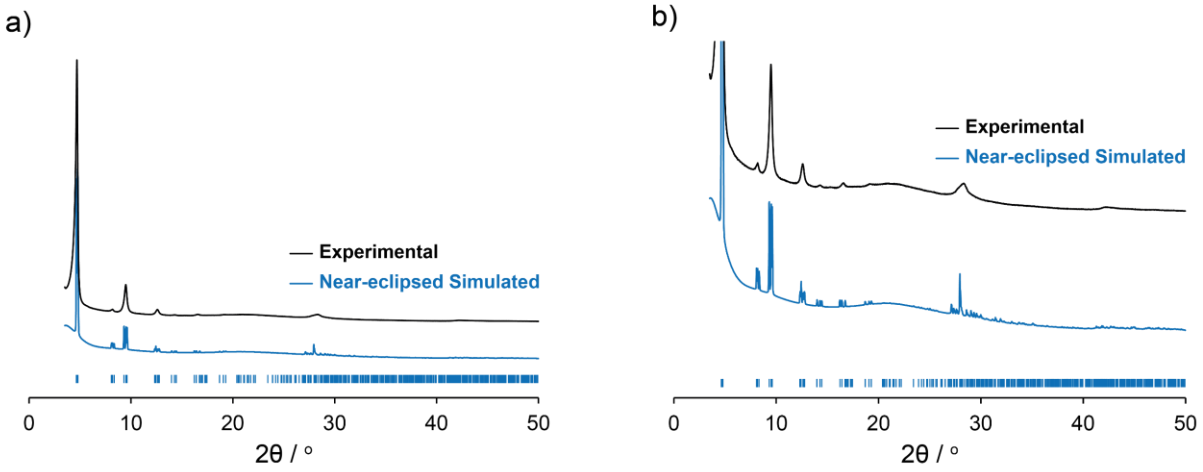

Figure S2: a) Comparison of the experimentally obtained PXRD pattern from $Cu_3(HHTP)_2$ synthesised in this work with the simulated PXRD pattern from $Cu_3(HHTP)_2$ with a near-eclipsed C-centred monoclinic crystal structure (space group = C2), in which the 2D layers of $Cu_3(HHTP)_2$ are displaced from eclipsed with a constant shift. Note the amorphous peak at $2\theta \approx 21$ ° is likely due to the borosilicate glass capillary tube.[6] b) A zoomed in view of the comparison for better visibility. Note that this data was of insufficient quality for a Rietveld refinement.

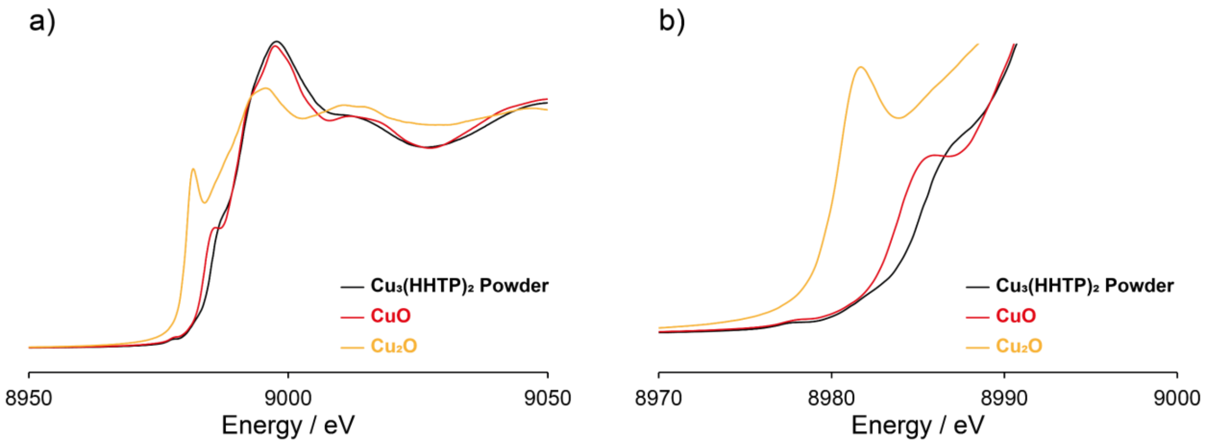

Figure S3: a) Cu K-edge XANES spectra of $Cu_3(HHTP)_2$ powder and standard compounds (CuO and Cu_2O). b) Expansion of the spectra around the pre-edge region shows little evidence for the presence of Cu(I) in $Cu_3(HHTP)_2$ due to the strong correlation between the absorption edge energy of $Cu_3(HHTP)_2$ and CuO and the absence of a peak at ca. 8981 eV, indicative of Cu(I). This strongly suggests that the major oxidation state in $Cu_3(HHTP)_2$ as synthesised in this work is Cu(II).

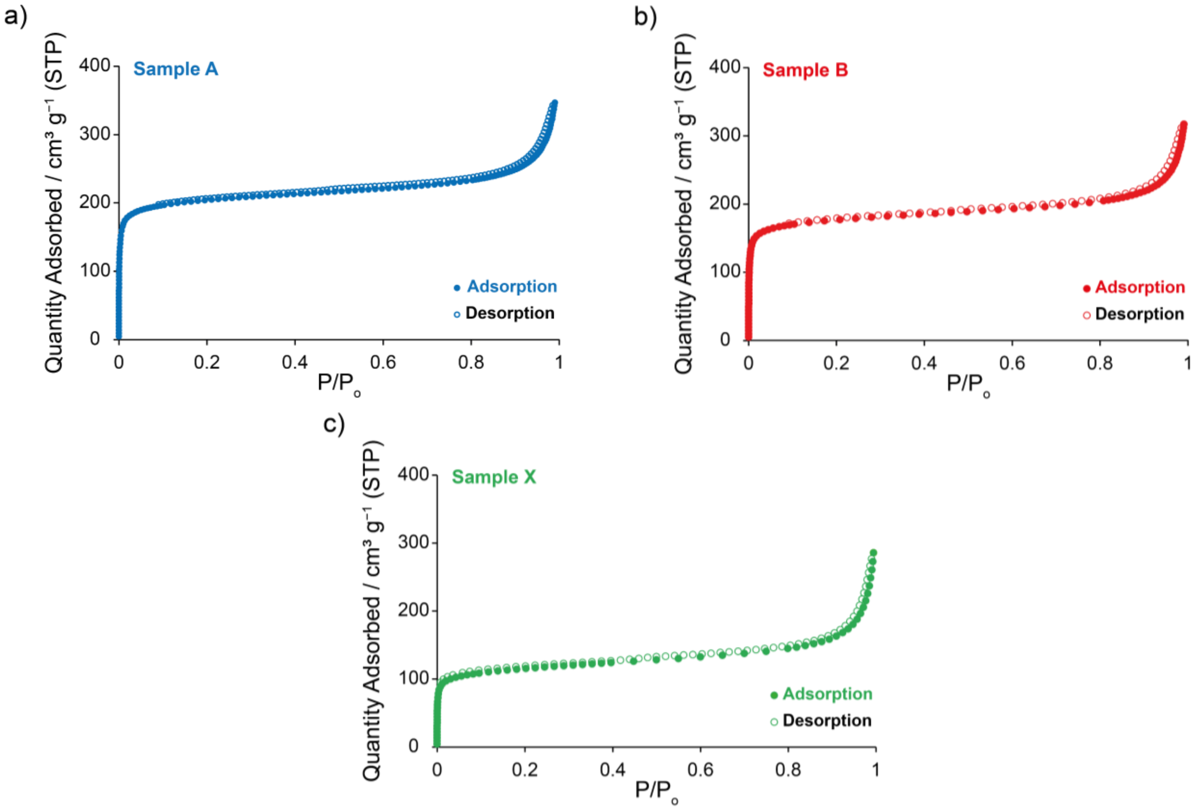

Figure S4: N_2 adsorption and desorption isotherms collected at 77 K for three powder samples of $Cu_3(HHTP)_2$. BET areas were calculated from the adsorption isotherms using Rouquerol's updated criteria implemented in BETSI. BET areas of 794 m^2 g^{-1}, 685 m^2 g^{-1}, and 435 m^2 g^{-1} were calculated for samples A, B and X, respectively. Samples A and B were washed as described above, whereas sample X was soaked in water (100 mL, 12 h), ethanol (100 mL, 3 h), and acetone (100 mL, 3 h) following the washing process. See Table S3 and the Appendix for more detail.

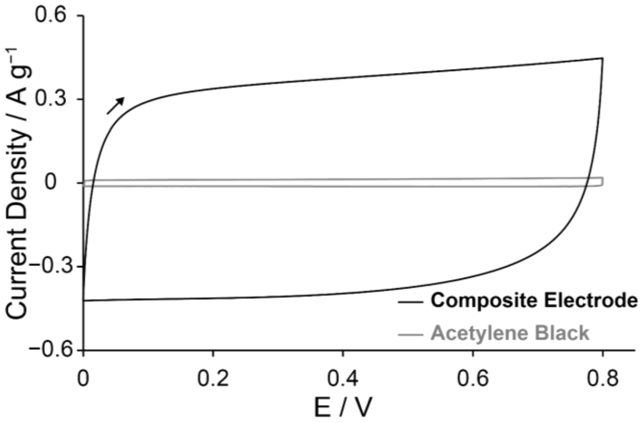

Figure S5: Comparison of cyclic voltammograms (CVs) obtained at a scan rate of 10 mV s^{-1} for symmetric EDLCs constructed using $Cu_3(HHTP)_2$ composite and acetylene black film electrodes, both with 1 M NEt_4BF_4 in acetonitrile electrolyte. From this, acetylene black has a calculated specific capacitance, C_g, of 2 – 3 F g^{-1}, and therefore has a negligible contribution to the capacitance of the $Cu_3(HHTP)_2$ composite electrodes.

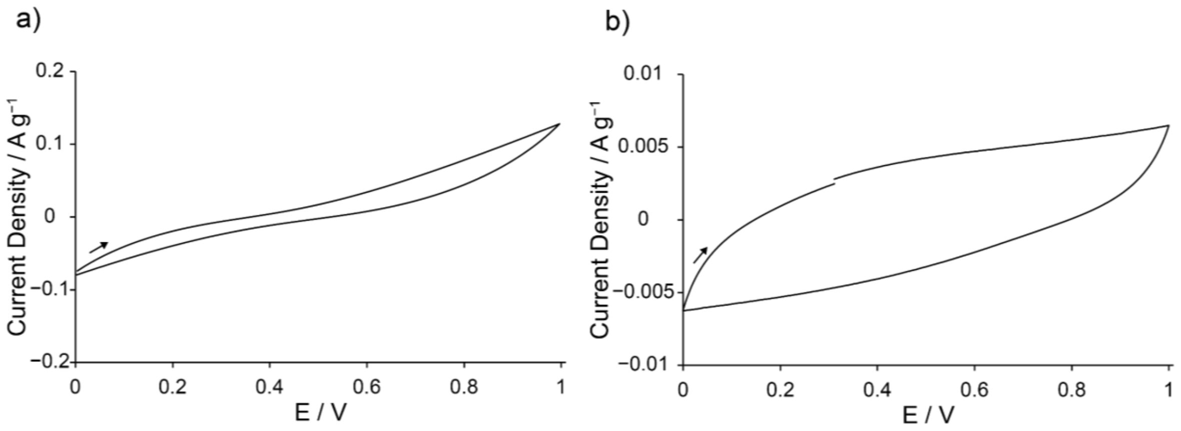

Figure S6: CVs obtained at scan rates of a) 10 mV s^{-1}; b) 0.1 mV s^{-1} from a symmetric EDLC constructed using $Cu_3(HHTP)_2$ electrodes with no conductive additive with 1 M NEt_4BF_4 in acetonitrile electrolyte. This demonstrates the highly resistive nature of this EDLC and the need for very low scan rates and current densities to obtain capacitive performance when the conductive additive is excluded.

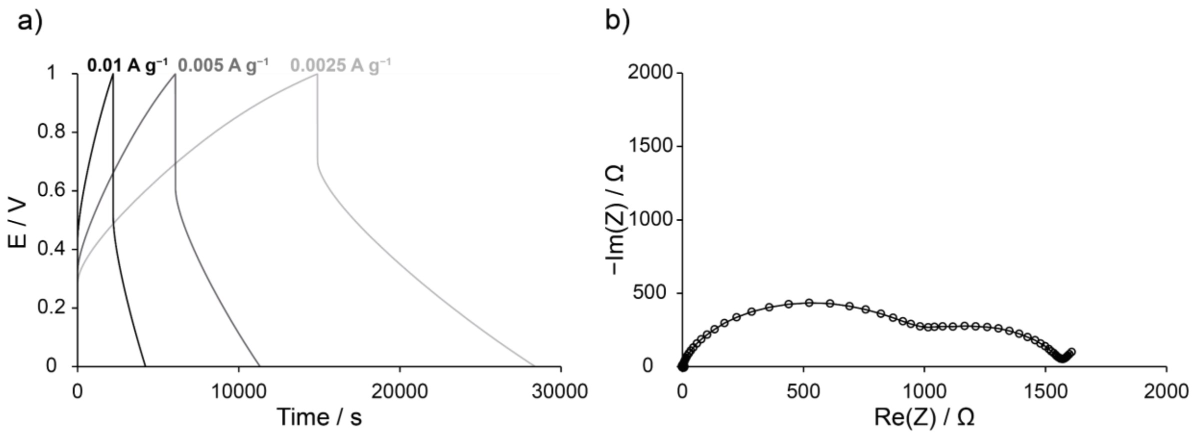

Figure S7: a) Galvanostatic charge-discharge (GCD) profiles at a variety of very low current densities from a symmetric EDLC constructed using $Cu_3(HHTP)_2$ electrodes with no conductive additive. b) Nyquist plot from electrochemical impedance spectroscopy (EIS) on the same cell further demonstrates the high resistance. From the voltage drop at the start of the GCD profiles, an *ESR* of approximately 10,000 – 15,000 Ω was calculated. This highlights the need for a conductive additive in the films. The specific capacitance, C_g, for this cell was calculated as 114 F g^{-1} at 0.0025 A g^{-1}.

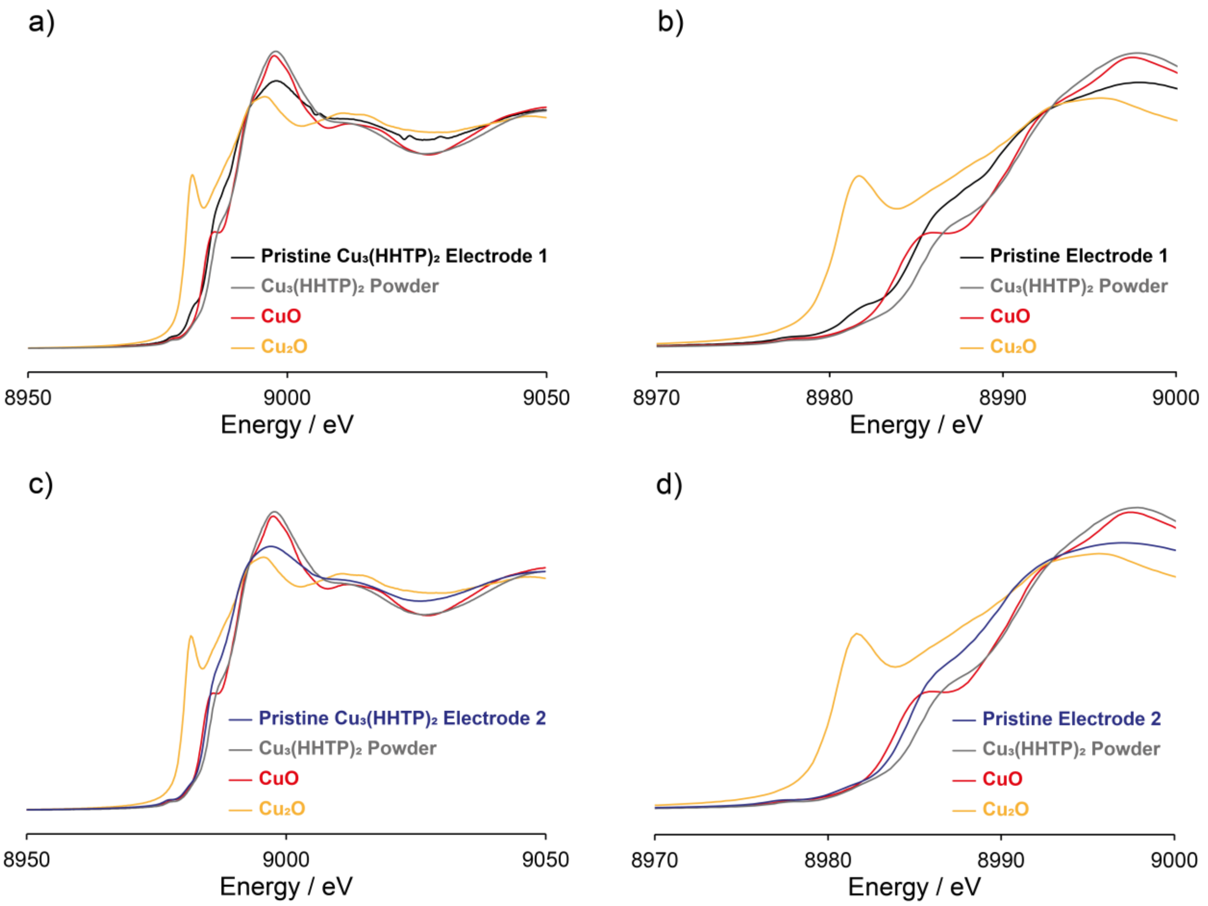

Figure S8: a), c) Cu K-edge XANES spectra of pristine (uncycled) $Cu_3(HHTP)_2$ film electrodes soaked in NEt_4BF_4 in acetonitrile compared with both $Cu_3(HHTP)_2$ powder and Cu(I) and Cu(II) standard compounds. b), d) Expansion of the spectra around the edge region shows evidence for the presence of Cu(I) in the pristine films due to a shift of the absorption edge to lower energies and the appearance of an inflection at ca. 8981 eV, due to the 1s to 4p transition of Cu(I). This suggests some formation of Cu(I) during the film making process, although the amount varied between pristine film samples. Note these films were produced from different samples of $Cu_3(HHTP)_2$.

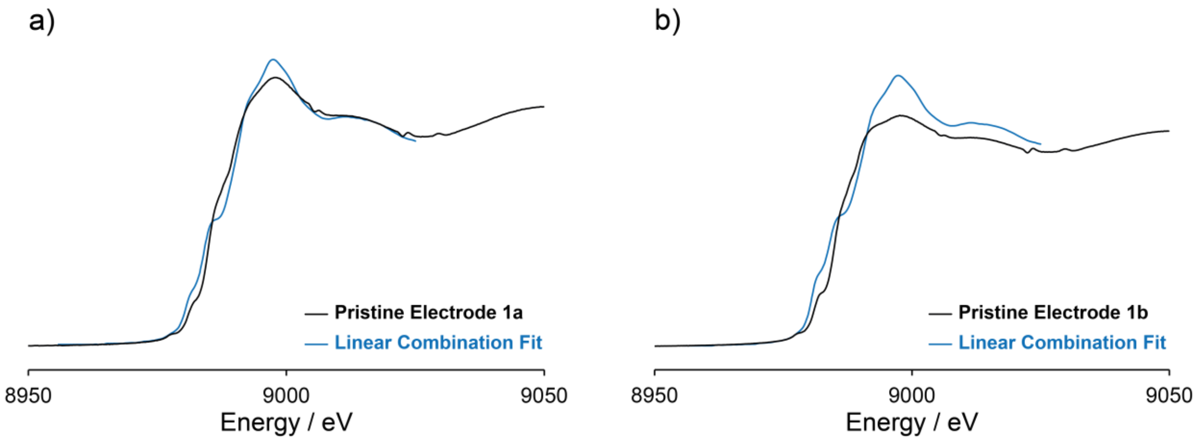

Figure S9: Linear combination fits of two Cu K-edge XANES spectra of pristine (uncycled) $Cu_3(HHTP)_2$ film electrodes with Cu(I) and Cu(II) standard compounds. From these fits, a Cu(I) to Cu(II) ratio of approximately 1 : 4 was calculated. However, the low quality of the fits, as the standards do not completely represent the sample and there may be other Cu environments present, impacts the reliability of these results. See Table S2 for more detail.

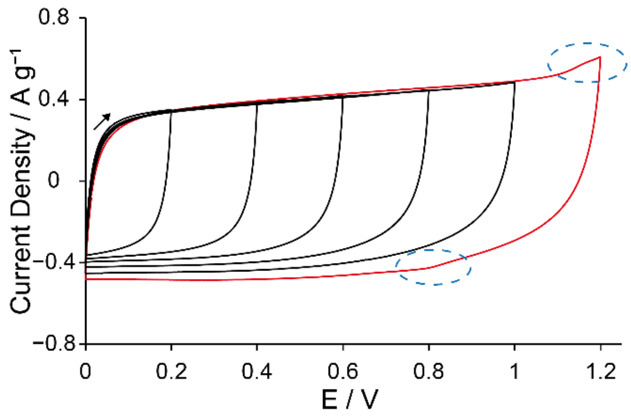

Figure S10: CVs obtained at a scan rate of 10 mV s^{-1} with increasing final cell voltages of 0.2, 0.4, 0.6, 0.8, 1.0 and 1.2 V for a symmetric EDLC made with $Cu_3(HHTP)_2$ composite electrodes and 1 M NEt_4BF_4 in acetonitrile electrolyte. CVs up to 1.0 V (black) show predominantly double-layer capacitive behaviour whereas distinct faradaic processes, centred at ca. 1.1 V, are observed upon cycling to higher final voltages (red - forward and reverse faradaic processes circled).

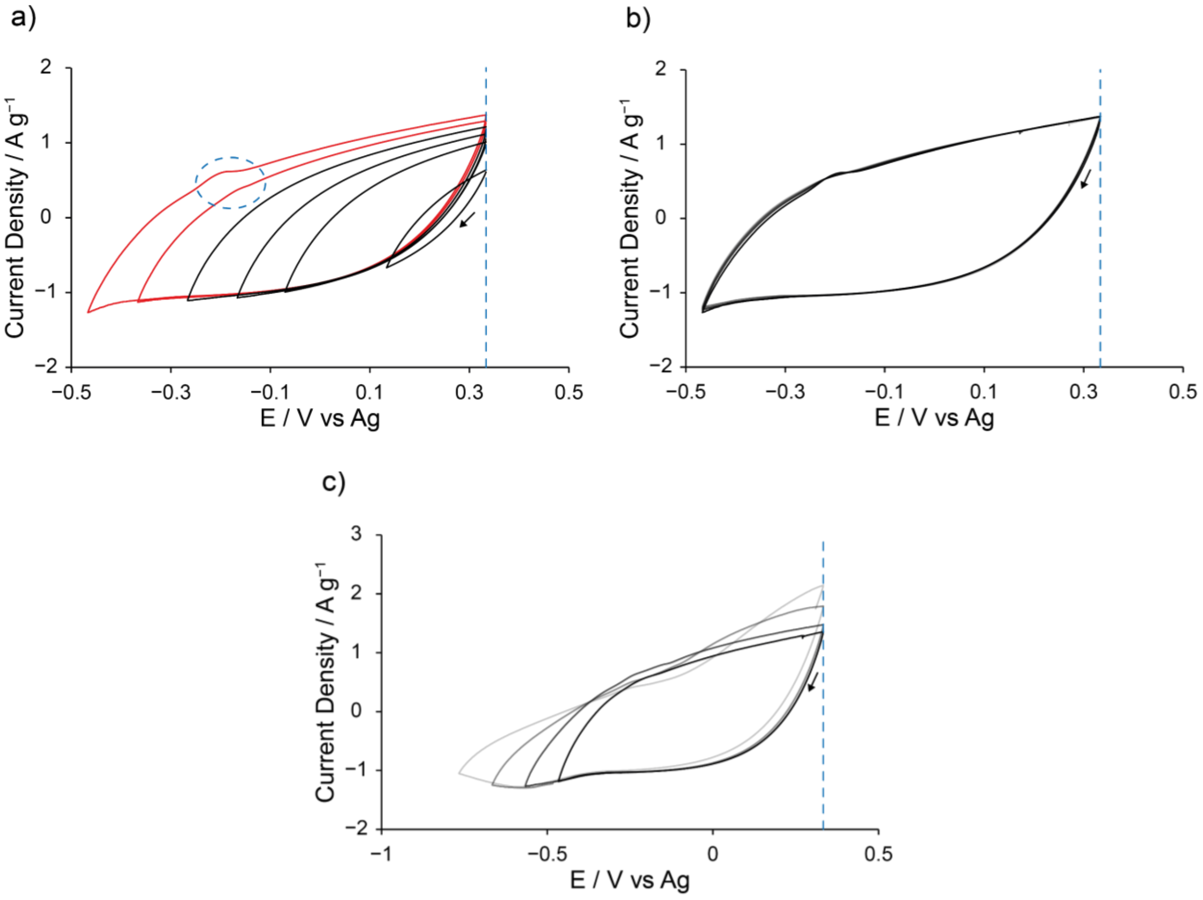

Figure S11: CVs obtained at a scan rate of 10 mV s^{-1} from a $Cu_3(HHTP)_2$ composite electrode in a three-electrode cell with 1 M NEt_4BF_4 in acetonitrile electrolyte. a) CVs with increasingly more negative final potentials from the open circuit voltage of 0.33 V (blue dotted line). CVs up to −0.27 V (black) show predominantly double-layer capacitive behaviour whereas distinct faradaic processes are observed upon cycling to higher final potentials (red with reverse faradaic peaks circled). b) Five CV cycles up to −0.47 V, showing only small irreversibility of the faradaic process at this potential. c) CVs with increasingly more negative final potentials above −0.47 V show increased faradaic activity. Note all potentials are referenced relative to Ag.

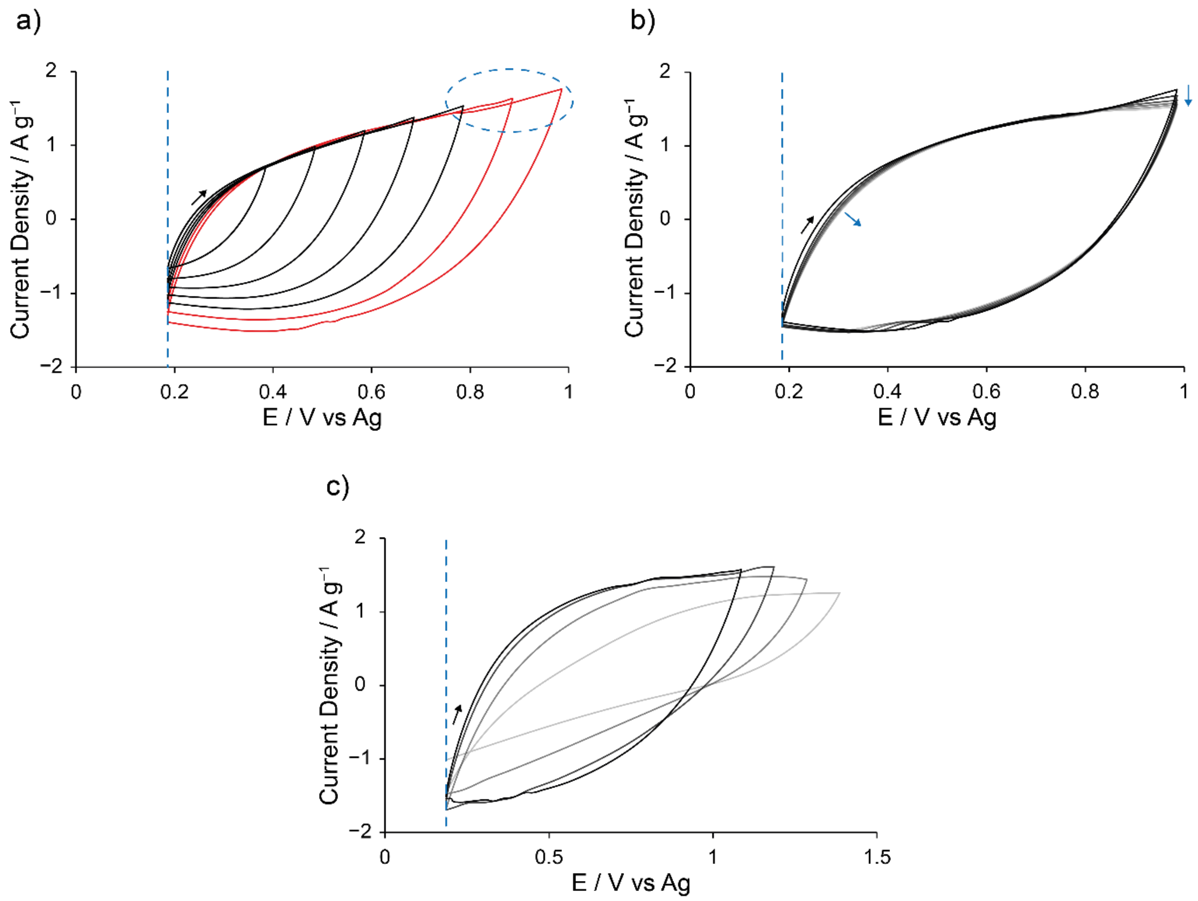

Figure S12: CVs obtained at a scan rate of 10 mV s^{-1} from a $Cu_3(HHTP)_2$ composite electrode in a three-electrode cell with 1 M NEt_4BF_4 in acetonitrile electrolyte. a) CVs with increasingly more positive final potentials from the open circuit voltage of 0.19 V (blue dotted line). CVs up to 0.79 V (black) show predominantly double-layer capacitive behaviour whereas distinct faradaic processes are observed upon cycling to higher final potentials (red with forward faradaic peaks circled). b) Five CV cycles up to 0.99 V, showing irreversibility of the faradaic process and loss of capacitance at this potential. c) CVs with increasingly more positive final potentials above 0.99 V show rapid capacitance loss. Note all potentials are referenced relative to Ag.

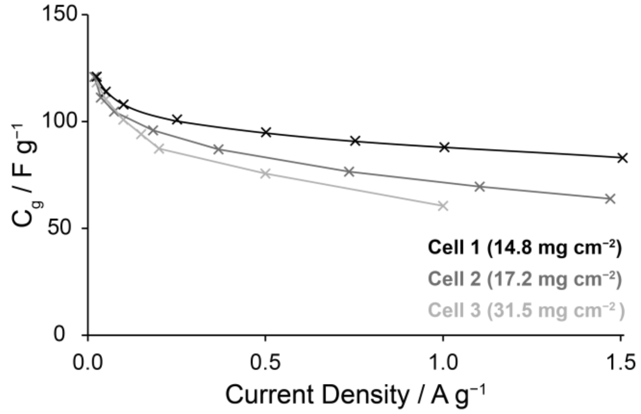

Figure S13: Specific capacitance, C_g, versus current density curves for three independent symmetric EDLCs, all constructed with $Cu_3(HHTP)_2$ composite electrodes and 1 M NEt_4BF_4 in acetonitrile electrolyte, with different electrode areal mass loadings. All EDLCs display similar C_g values at low current densities (between 110 – 114 F g^{-1} at 0.04 – 0.05 A g^{-1}) while EDLCs with greater mass loadings show a more rapid decrease in C_g with increasing current density. See Table S2 for a full comparison of the performances of these three EDLCs.

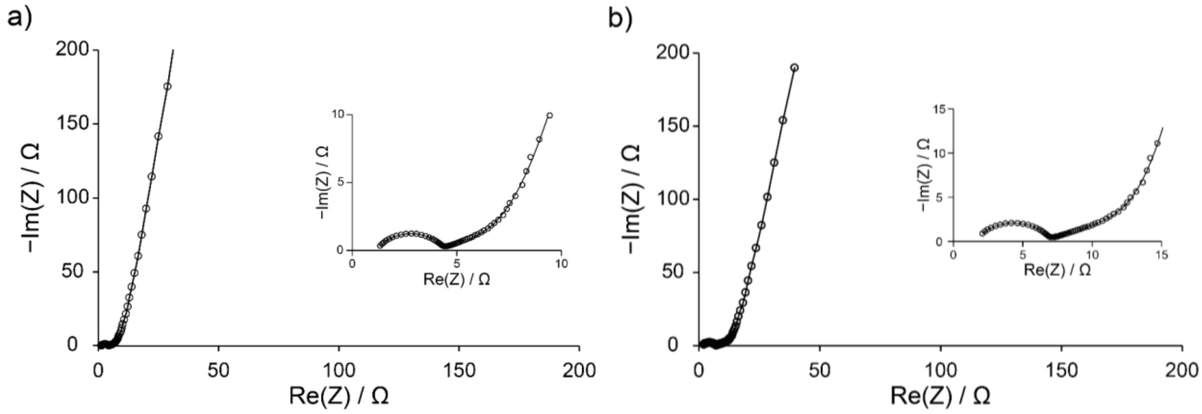

Figure S14: Nyquist plots for two symmetric $Cu_3(HHTP)_2$ composite EDLCs, a) Cell 1; b) Cell 2, obtained from electrochemical impedance spectroscopy (EIS). Both Nyquist plots exhibit a semi-circular response at high frequencies, indicative of contact resistances, and a linear response at low frequencies, which is typical of EDLCs. Both exhibit a Warburg transition at a knee frequency of between 0.8 – 2.9 Hz. This suggests that there is diffusion-limited transport of electrolyte ions into the electrodes. Equivalent series resistances (*ESR*s) were calculated from the above by extrapolating the low-frequency response onto the real (*Re(Z)*) axis. See Table S2 for full details.

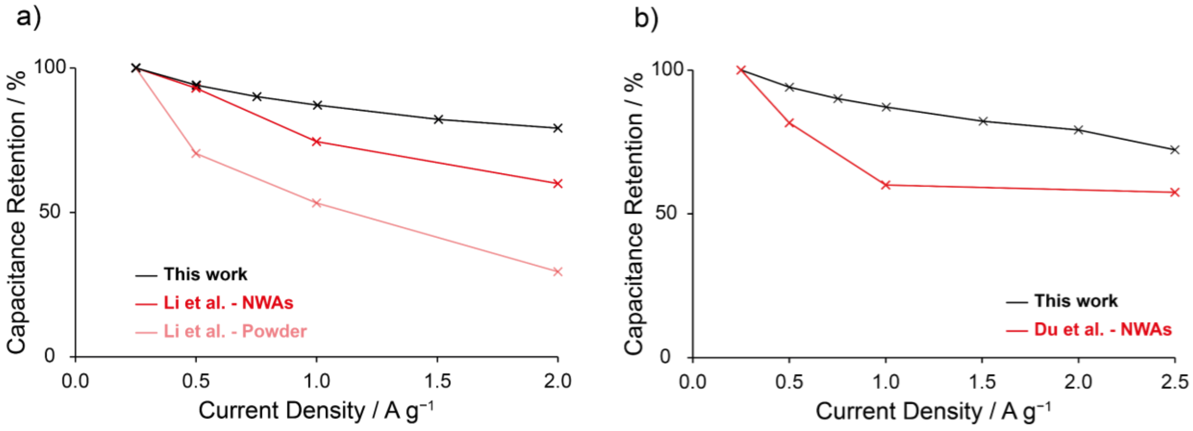

Figure S15: Comparison of capacitance retention versus current density plots from a symmetric $Cu_3(HHTP)_2$ composite EDLC produced in this work with a) solid-state supercapacitors assembled by Li et al. using both $Cu_3(HHTP)_2$ powder and NWA electrodes[7]; b) an aqueous EDLC assembled by Du et al. using $Cu_3(HHTP)_2$ NWA electrodes.[8]

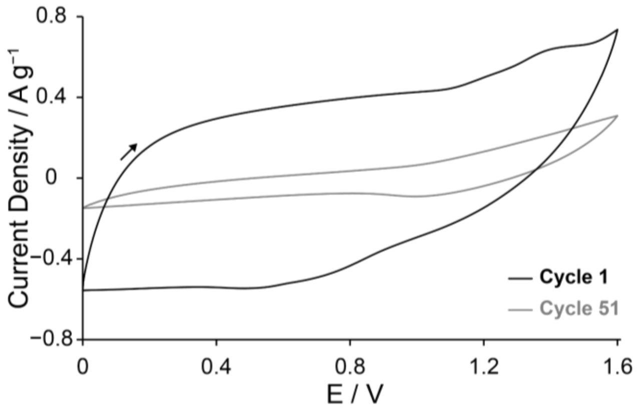

Figure S16: CVs obtained at a scan rate of 10 mV s^{-1} up to a final voltage of 1.6 V. Rapid loss of capacitance over 51 cycles is observed, showing the very low stability of $Cu_3(HHTP)_2$ when cycling up to this cell voltage in symmetric EDLCs with 1 M NEt_4BF_4 in acetonitrile electrolyte.

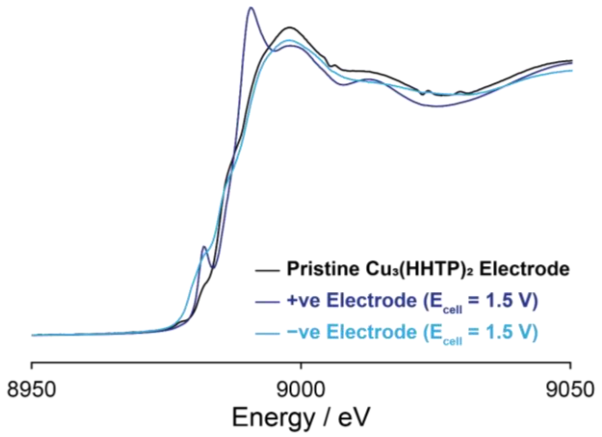

Figure S17: Cu K-edge XANES spectra of $Cu_3(HHTP)_2$ composite electrodes from a symmetric EDLC assembled with 1 M NEt_4BF_4 in acetonitrile electrolyte held at a cell voltage of 1.5 V for 1 h prior to disassembly. The shift of the absorption edge to a lower energy, in addition to an increase in intensity of the feature at ca. 8981 eV, in the XANES spectrum of the negative electrode is indicative of reduction to form Cu(I). The appearance of the same feature in the positive electrode without an associated shift in the absorption edge to lower energies is indicative of a change in the coordination environment around the Cu nodes to a lower symmetry. These observations hint at potential degradation mechanisms, although further work is required to identify the degradation mechanisms in more detail. See the Discussion above for more detailed interpretation of the results.

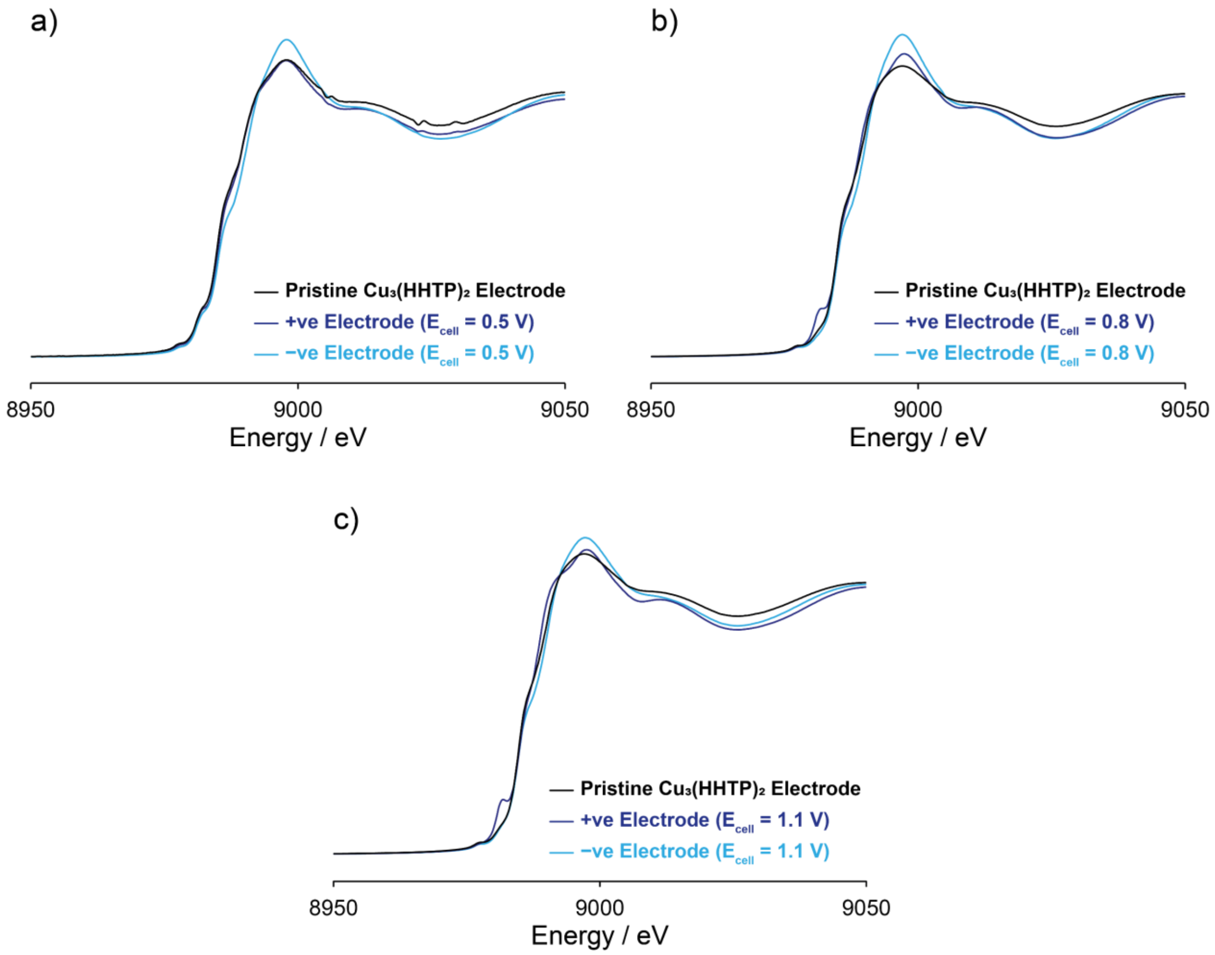

Figure S18: Cu K-edge XANES spectra of $Cu_3(HHTP)_2$ composite electrodes from symmetric EDLCs, assembled with 1 M NEt_4BF_4 in acetonitrile electrolyte, held at a variety of different cell voltages for 1 h prior to disassembly. Spent electrodes from an EDLC held at 0.5 V prior to assembly show minimal change to the electrode structures relative to a pristine electrode from the same film. While the negative electrodes from EDLCs held at 0.8 and 1.1 V show minimal change, the appearance of a feature at ca. 8981 eV in the XANES spectra of the positive electrodes from these cells, without an associated shift in the absorption edge energy, is indicative of a change in the coordination environment around the Cu nodes to a lower symmetry. This indicates faradaic processes occur in the positive electrodes at these cell voltages.

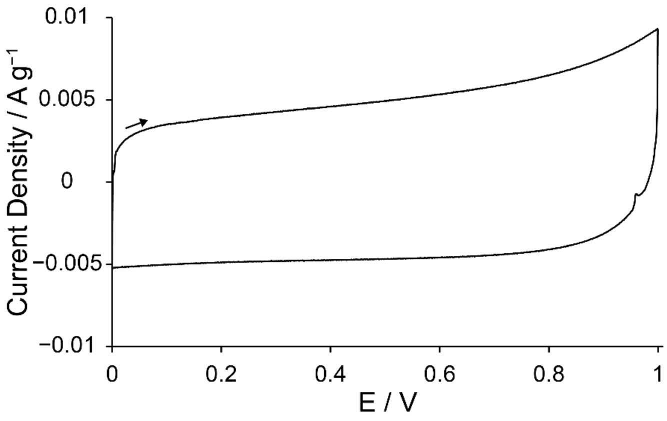

Figure S19: CV obtained at a scan rate of 0.1 mV s^{-1} up to 1 V for a symmetric EDLC made with $Cu_3(HHTP)_2$ composite electrodes and 1 M NEt_4BF_4 in acetonitrile electrolyte. At this low scan rate, some faradaic activity is observed on the forward scan after reaching a potential of ca. 0.8 V.

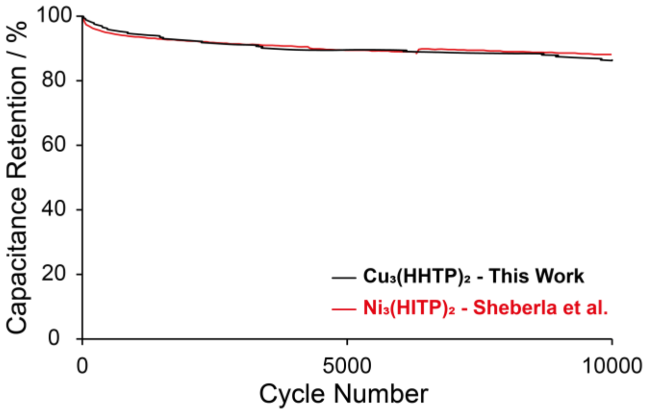

Figure S20: Comparison of capacitance retention versus cycle number plots for $Cu_3(HHTP)_2$ composite electrodes, when cycled between 0 – 1 V at 1 A g^{-1} in this work, with $Ni_3(HITP)_2$ electrodes, when cycled between 0 – 1 V at 2 A g^{-1} by Sheberla et al., over 10,000 cycles.[9] Cycling stability was measured for symmetric EDLCs with 1 M NEt_4BF_4 in acetonitrile electrolyte for both materials.

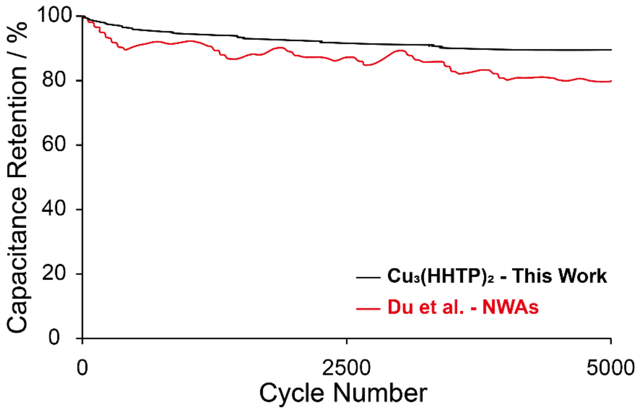

Figure S21: Comparison of capacitance retention versus cycle number plots for $Cu_3(HHTP)_2$ composite electrodes, when cycled between 0 – 1 V at 1 A g^{-1} in this work, and $Cu_3(HHTP)_2$ NWA-based electrodes, when cycled between 0 – 0.8 V at 5 A g^{-1} by Du et al., over 5,000 cycles. Cycling stability was measured for a symmetric EDLC with 1 M NEt_4BF_4 in acetonitrile electrolyte in this work, and for a symmetric EDLC with 1 M KCl in H_2O electrolyte by Du et al.[8]

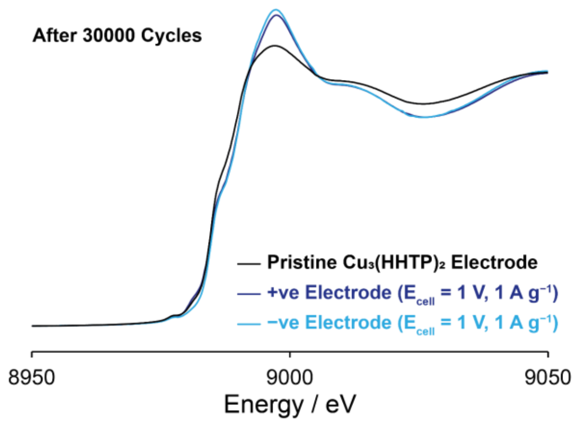

Figure S22: Cu K-edge XANES spectra of $Cu_3(HHTP)_2$ composite electrodes from a symmetric EDLC which was cycled between 0 – 1 V at 1 A g^{-1} for 30,000 cycles. The cell was held at 0 V for 1 h prior to disassembly. Minimal changes to the structure of both electrodes relative to a pristine electrode from the same film were observed following cycling, with no shift in the absorption edge energy and minimal increase in intensity of the feature at 8981 eV.

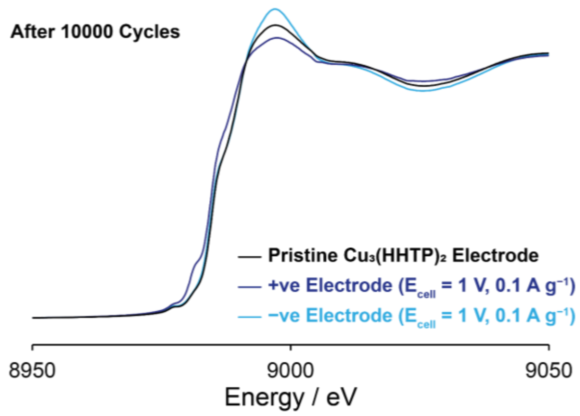

Figure S23: Cu K-edge XANES spectra of $Cu_3(HHTP)_2$ composite electrodes from a symmetric EDLC which was cycled between 0 – 1 V at 0.1 A g^{-1} for 10,000 cycles. The cell was held at 0 V for 1 h prior to disassembly. Significant change in the structure of the positive electrode was deduced by the shift in the absorption edge to a lower energy and the appearance of a feature at ca. 8981 eV. This indicates potential formation of Cu(I) along with a lowering of the symmetry of the Cu nodes. Little change in the structure of the negative electrode is observed.

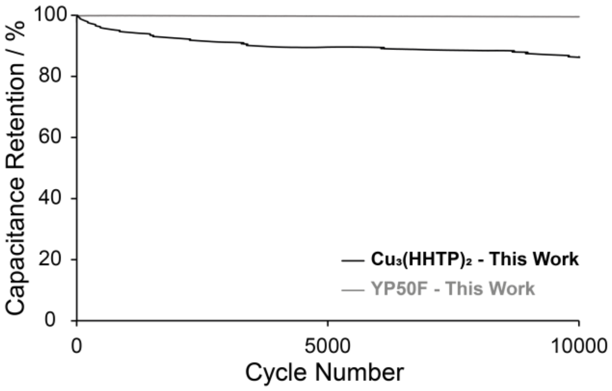

Figure S24: Comparison of capacitance retention versus cycle number plots for $Cu_3(HHTP)_2$ composite electrodes, when cycled between 0 – 1 V at 1 A g^{-1}, and YP50F activated carbon, when cycled between 0 – 2.5 V at a similar current density of 2 A g^{-1}, over 10,000 cycles. Cycling stability was measured for symmetric EDLCs with 1 M NEt_4BF_4 in acetonitrile electrolyte for both materials.

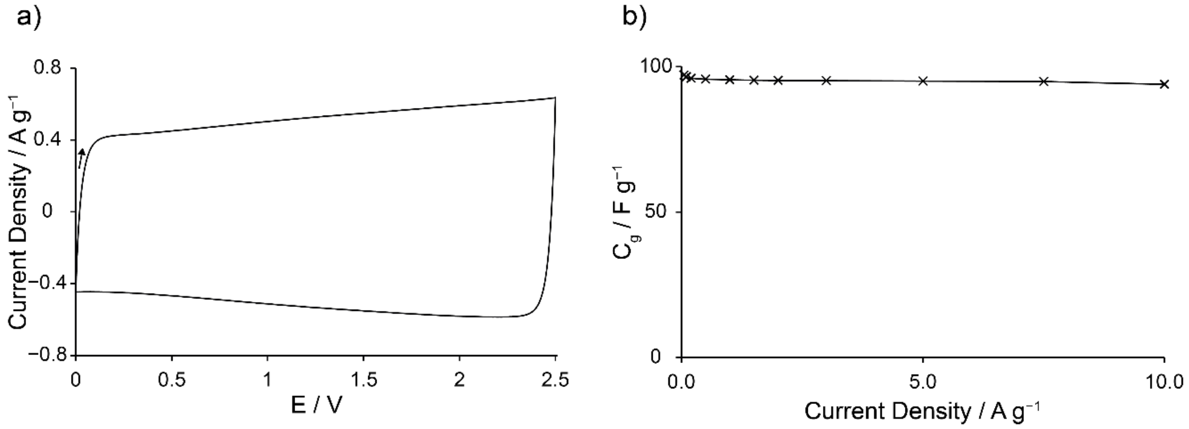

Figure S25: a) CV obtained at 10 mV s^{-1} up to 2.5 V; b) specific capacitance versus current density plot for a symmetric EDLC constructed with YP50F film electrodes and 1 M NEt_4BF_4 in acetonitrile electrolyte, when charged between 0 – 2.5 V. These demonstrate the wide voltage window and high capacitive performance of this commercially available activated carbon.

Table S1: Crystallographic Data for Simulated $Cu_3(HHTP)_2$ Structures

	Structure 1	Structure 2
Crystal System	Hexagonal (Eclipsed)	Monoclinic (Near-eclipsed)
Space Group	P622	C2
a / Å	21.50	21.18
b / Å	21.50	37.98
c / Å	3.20	3.31
α / º	90	90
β / º	90	92.36
γ / º	120	90
Volume of Unit Cell / $Å^3$	1281	2661

Table S2: Cu K-edge XANES Linear Combination Fittings

Pristine $Cu_3(HHTP)_2$ composite electrode	Weight CuO	Weight $Cu(OAc)_2$	Weight Cu_2O	Weight CuOAc	Total weight of Cu(II)	Total weight of Cu(I)	R-factor
1a	0.697	0.077	0.164	0.061	0.774	0.226	0.0084842
1b	0.615	0.162	0.223	0	0.777	0.223	0.0057456

Table S3: Performance Comparison of Three Independent EDLCs

Cell	Total mass of $Cu_3(HHTP)_2$ in electrodes / mg	Mass loading of $Cu_3(HHTP)_2$ per electrode / mg cm^{-2}	C_g / F g^{-1} (GCD, 0.04 – 0.05 A g^{-1}, 0 – 1 V)	*ESR* / Ω (GCD, 0.04 – 0.05 A g^{-1}, 0 – 1 V)	*ESR* / Ω (EIS)
1	9.4	14.8	114	7.6	6.6
2	10.9	17.2	111	12.4	13.6
3	19.9	31.4	110	17.0	17.3

Table S4: Comparison of Areal Capacitance of EDLCs Made from Different Batches of $Cu_3(HHTP)_2$

$Cu_3(HHTP)_2$ Sample	Cell	BET Area / m^2 g^{-1}	C_g / F g^{-1} (GCD, 0.04 – 0.05 A g^{-1}, 0.8/1 V)	C_a / μF cm^{-2} (GCD, 0.04 – 0.05 A g^{-1}, 0.8/1 V)
A	2	794	111	14.0
B	4	685	110	16.0
X*	5	435	100**	23.1**

*Sample X was soaked following washing.

**Note a stable double-layer voltage window of 0.8 V was determined and used for cell 5.

Appendix

BETSI Analysis & Regression Diagnostics for Sample A

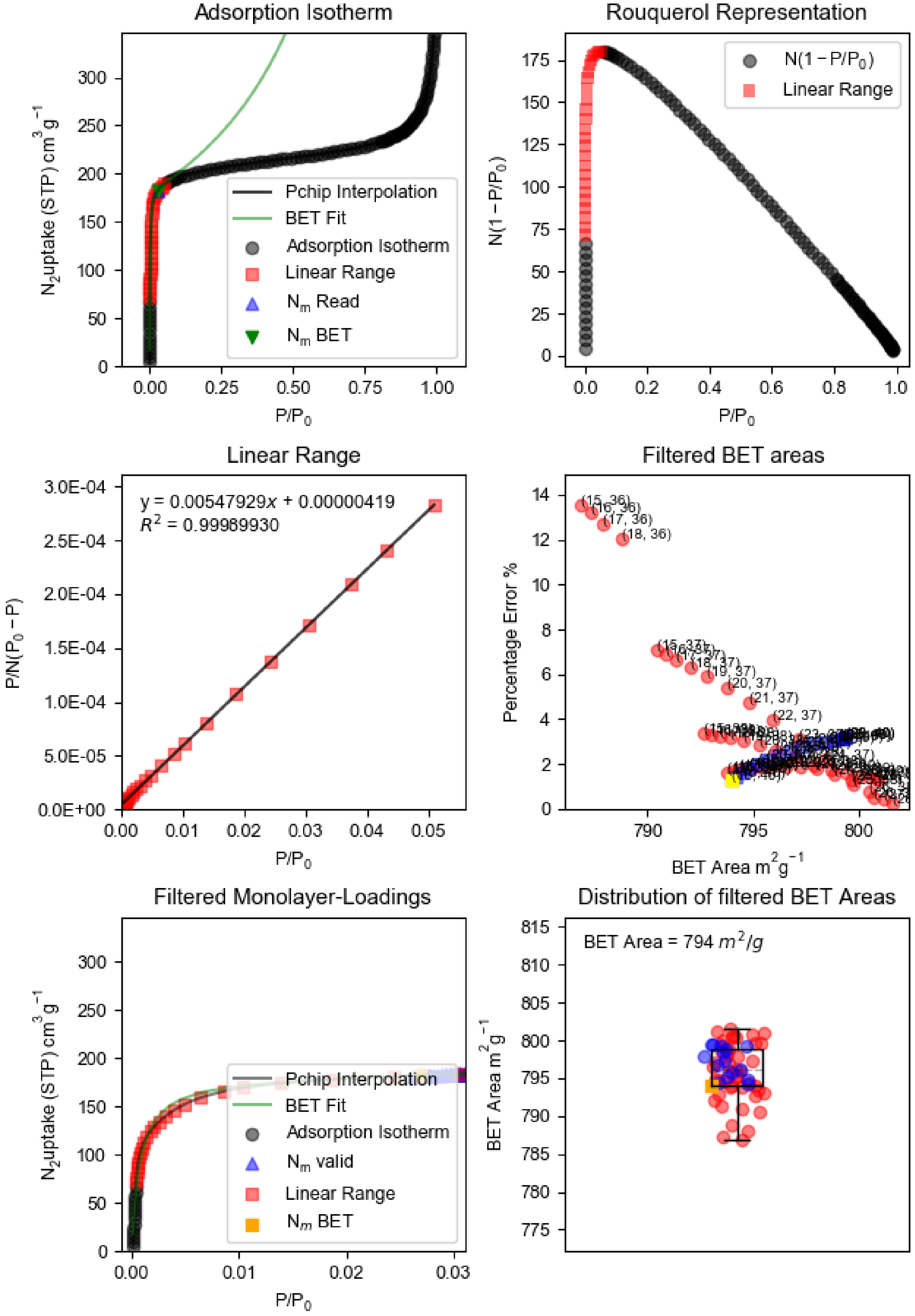

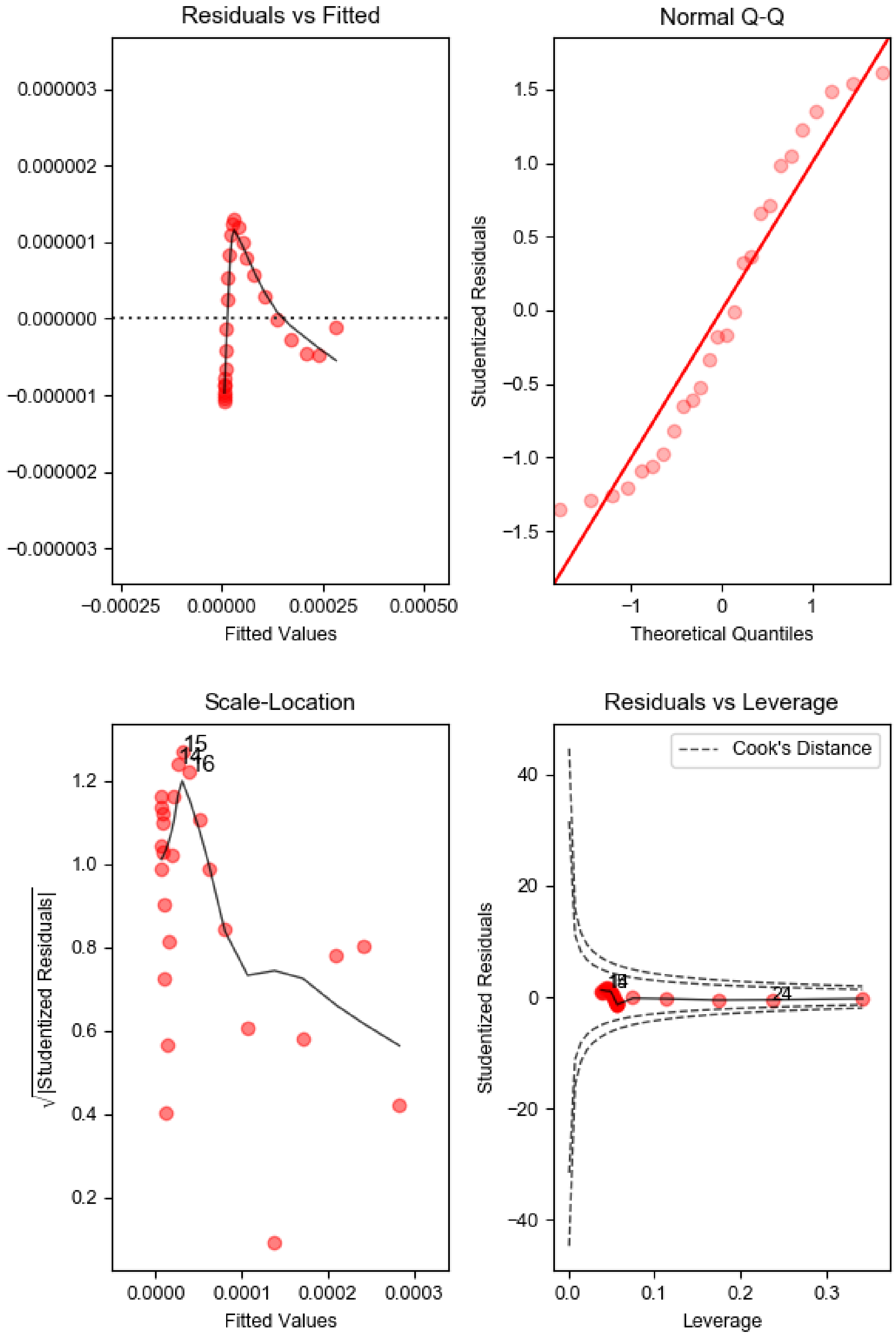
Residuals vs Fitted
Fitted Values
Normal Q-Q
Studentized Residuals
Theoretical Quantiles
Scale-Location
√|Studentized Residuals|
Fitted Values
Residuals vs Leverage
Cook's Distance
Studentized Residuals
Leverage

BETSI Analysis & Regression Diagnostics for Sample B

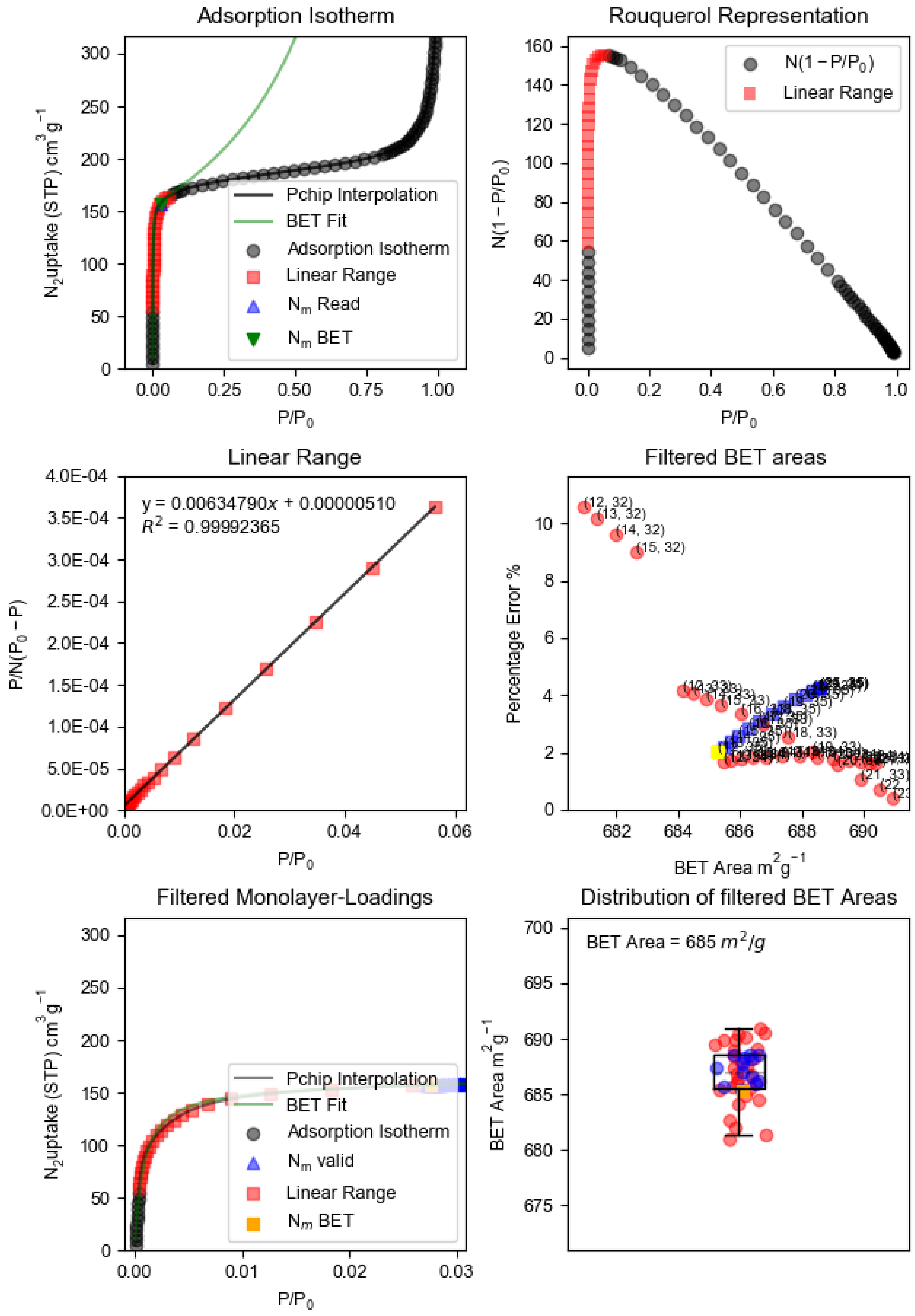

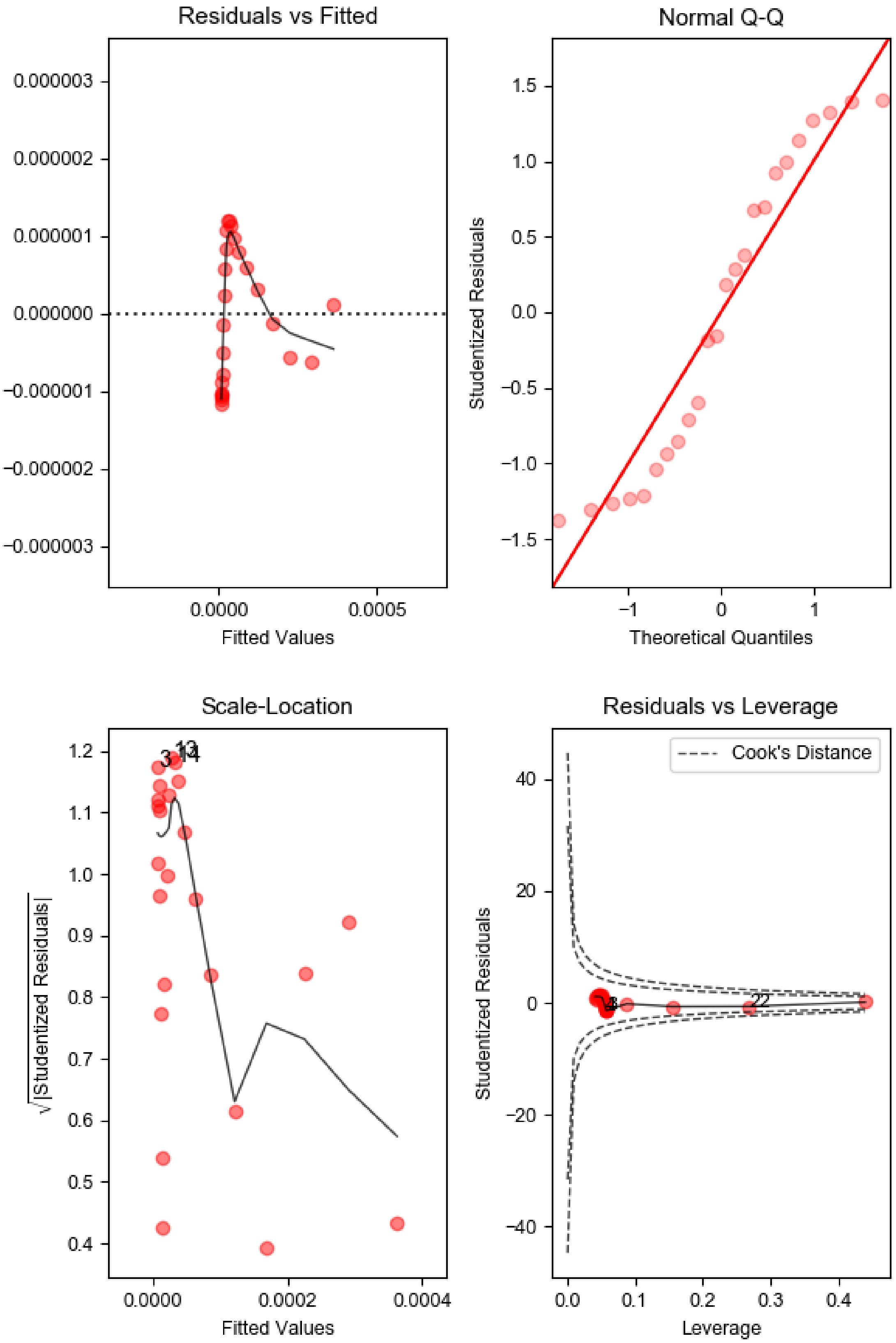
Residuals vs Fitted
0.000003
0.000002
0.000001
0.000000
−0.000001
−0.000002
−0.000003
0.0000
0.0005
Fitted Values
Normal Q-Q
1.5
1.0
0.5
0.0
−0.5
−1.0
−1.5
Studentized Residuals
−1
0
1
Theoretical Quantiles
Scale-Location
1.2
1.1
1.0
0.9
0.8
0.7
0.6
0.5
0.4
√|Studentized Residuals|
0.0000
0.0002
0.0004
Fitted Values
Residuals vs Leverage
Cook's Distance
40
20
0
−20
−40
Studentized Residuals
0.0
0.1
0.2
0.3
0.4
Leverage

BETSI Analysis & Regression Diagnostics for Sample X

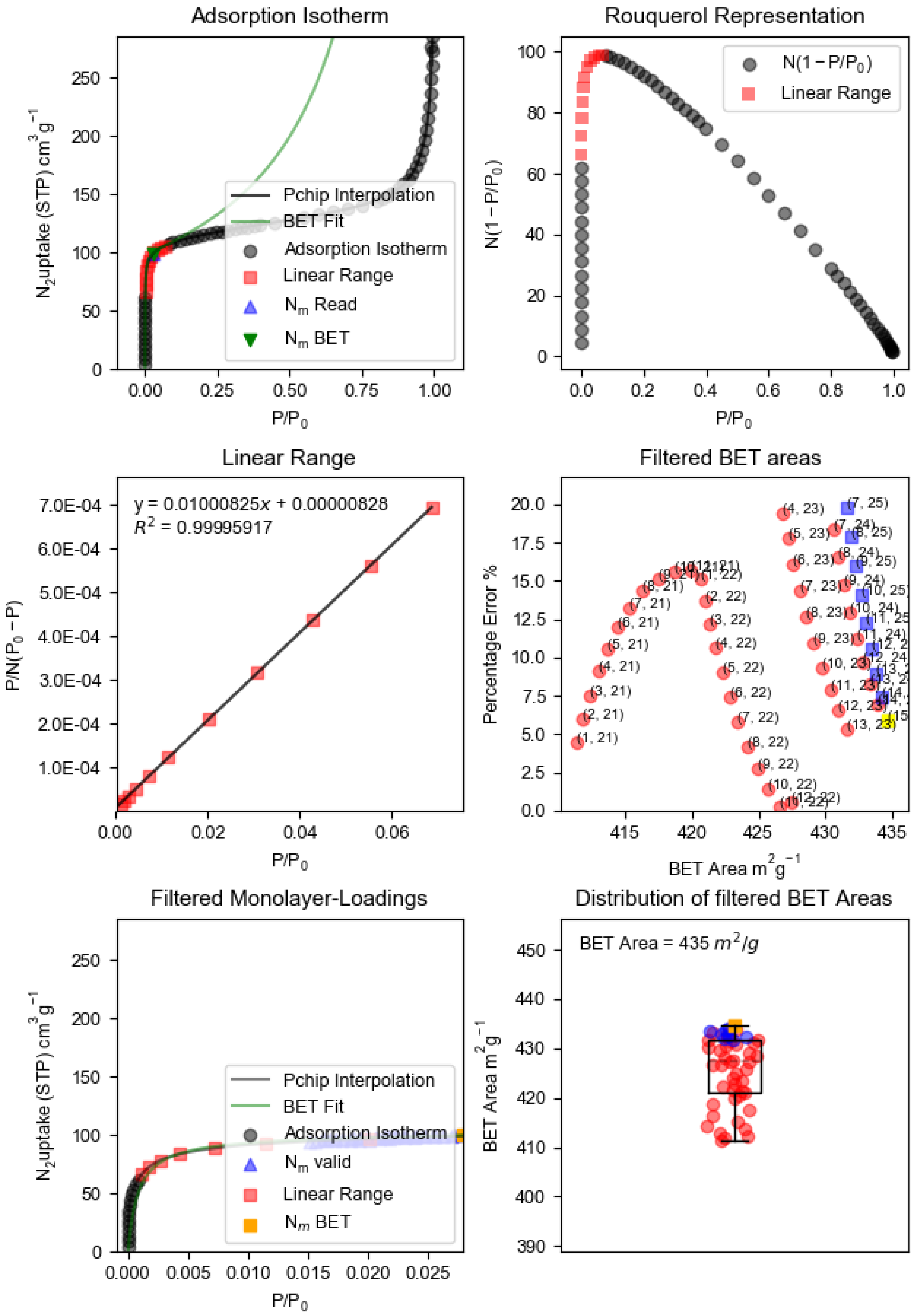

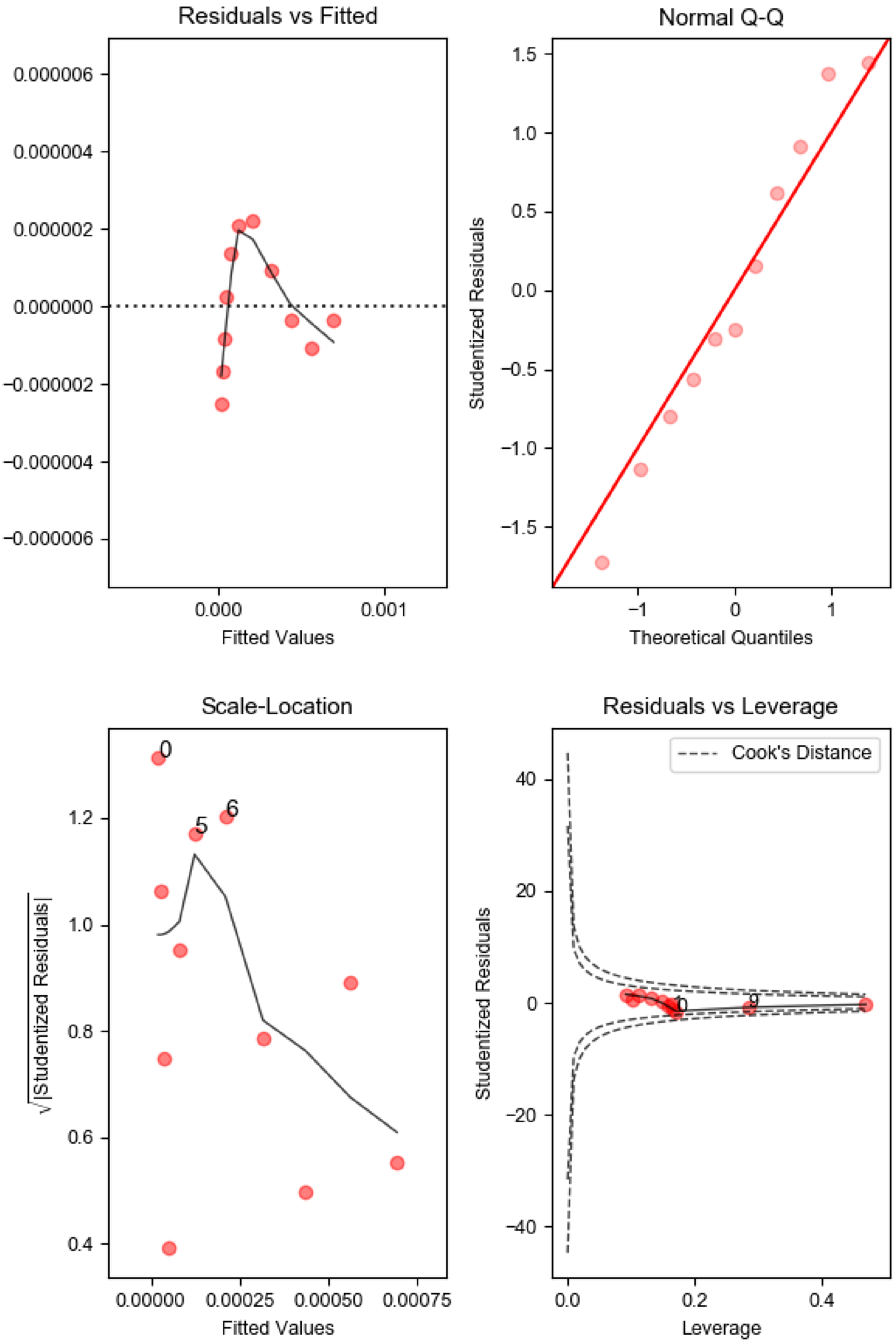
Residuals vs Fitted
0.000006
0.000004
0.000002
0.000000
−0.000002
−0.000004
−0.000006
0.000
0.001
Fitted Values
Normal Q-Q
1.5
1.0
0.5
0.0
−0.5
−1.0
−1.5
−1
0
1
Studentized Residuals
Theoretical Quantiles
Scale-Location
0
5
6
1.2
1.0
0.8
0.6
0.4
√|Studentized Residuals|
0.00000
0.00025
0.00050
0.00075
Fitted Values
Residuals vs Leverage
Cook's Distance
40
20
0
−20
−40
Studentized Residuals
0.0
0.2
0.4
Leverage

References

1 M. Sano, S. Komorita and H. Yamatera, *Inorg. Chem.*, 1992, **31**, 459–463.

2 L.-S. Kau, D. J. Spira-Solomon, J. E. Penner-Hahn, K. O. Hodgson and E. I. Solomon, *J. Am. Chem. Soc.*, 1987, **109**, 6433–6442.

3 N. C. Tomson, K. D. Williams, X. Dai, S. Sproules, S. Debeer, T. H. Warren and K. Wieghardt, *Chem. Sci.*, 2015, **6**, 2474–2487.

4 J. Rudolph and C. R. Jacob, *Inorg. Chem.*, 2018, **57**, 10591–10607.

5 J. L. DuBois, P. Mukherjee, T. D. P. Stack, B. Hedman, E. I. Solomon and K. O. Hodgson, *J. Am. Chem. Soc.*, 2000, **122**, 5775–5787.

6 G. Shao, X. Wu, Y. Kong, S. Cui, X. Shen, C. Jiao and J. Jiao, *Surf. Coat. Technol.*, 2015, **270**, 154–163.

7 W. H. Li, K. Ding, H. R. Tian, M. S. Yao, B. Nath, W. H. Deng, Y. Wang and G. Xu, *Adv. Funct. Mater.*, 2017, **27**, 1702067

8 X. Du, J. Zhang, H. Wang, Z. Huang, A. Guo, L. Zhao, Y. Niu, X. Li, B. Wu and Y. Liu, *Mater. Chem. Front.*, 2020, **4**, 243–251.

9 D. Sheberla, J. C. Bachman, J. S. Elias, C. J. Sun, Y. Shao-Horn and M. Dincă, *Nat. Mater.*, 2017, **16**, 220–224.
